# Spatio-Temporal Variation in Age Structure and Abundance of the Endangered Snail Kite: Pooling across Regions Masks a Declining and Aging Population

**DOI:** 10.1371/journal.pone.0162690

**Published:** 2016-09-28

**Authors:** Brian E. Reichert, William L. Kendall, Robert J. Fletcher, Wiley M. Kitchens

**Affiliations:** 1 Department of Wildlife Ecology and Conservation, University of Florida, Gainesville, FL, United States of America; 2 U.S. Geological Survey, Colorado Cooperative Fish and Wildlife Research Unit, Fort Collins, CO, United States of America; University of Arkansas Fayetteville, UNITED STATES

## Abstract

While variation in age structure over time and space has long been considered important for population dynamics and conservation, reliable estimates of such spatio-temporal variation in age structure have been elusive for wild vertebrate populations. This limitation has arisen because of problems of imperfect detection, the potential for temporary emigration impacting assessments of age structure, and limited information on age. However, identifying patterns in age structure is important for making reliable predictions of both short- and long-term dynamics of populations of conservation concern. Using a multistate superpopulation estimator, we estimated region-specific abundance and age structure (the proportion of individuals within each age class) of a highly endangered population of snail kites for two separate regions in Florida over 17 years (1997–2013). We find that in the southern region of the snail kite—a region known to be critical for the long-term persistence of the species—the population has declined significantly since 1997, and during this time, it has increasingly become dominated by older snail kites (> 12 years old). In contrast, in the northern region—a region historically thought to serve primarily as drought refugia—the population has increased significantly since 2007 and age structure is more evenly distributed among age classes. Given that snail kites show senescence at approximately 13 years of age, where individuals suffer higher mortality rates and lower breeding rates, these results reveal an alarming trend for the southern region. Our work illustrates the importance of accounting for spatial structure when assessing changes in abundance and age distribution and the need for monitoring of age structure in imperiled species.

## Introduction

It is well known that age structure influences population dynamics [1–4]. In conservation, reliable information on the relative number of individuals within each age class of a population is often needed for tools such as population viability analysis [5–7]. However, population age structure is dependent on demographic vital rates (survival and reproduction) that can differ between habitat types [8] and vary over time in response to environmental disturbances [9]. Consequently, conclusions from studies that ignore variation in age structure can be misleading [10,11]. Assessing age structure at multiple spatiotemporal scales provides valuable information that may be important for predicting the dynamics of populations of conservation concern [10].

Spatial variation in age structure can develop over time for at least two reasons. First, if recruitment is limited within a local population, then over time individuals will age and older individuals will disproportionately make up the local population in the absence of immigration [10]. Such situations may be problematic for conservation, particularly when senescence occurs. Second, spatial variation in age structure can also arise due to despotic behaviors (*sensu* [12], where older, dominant individuals tend to settle in higher-quality habitats and younger individuals settle in lower-quality habitats (e.g., [8,13,14]). In such situations, populations can be relatively stable [15]. Therefore, determining trends in local abundance can be important for interpreting the potential implications of spatial variation in age structure. Unfortunately, reliable information on local population size and the relative number of individuals in different age classes (or age distribution) is often lacking [10].

Here, we present an approach for simultaneously estimating age distribution and population size, thereby allowing to estimate both relative and absolute variation in age structure. This approach accounts for imperfect detection and variation in seasonal arrival and departure times (both temporal and age-dependent variation). We use this approach to understand population dynamics and spatio-temporal variation in age structure in a highly endangered population of snail kites (*Rostrhamus sociabilis*) in Florida.

**Fig 1:**
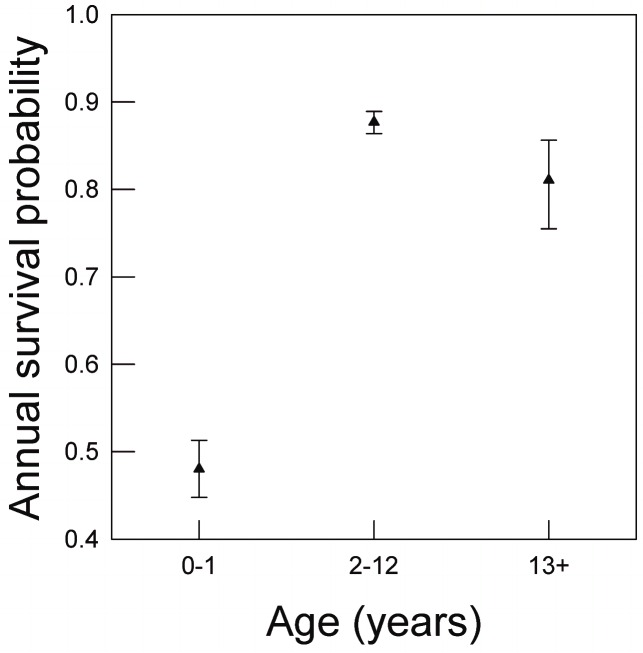
Age-class specific apparent annual range-wide survival of snail kites in Florida, modified from Reichert et al. [16]. Results from previous analyses reveal that adult survival declines significantly in snail kites beginning at age 13. Estimates are based on modeling encounter histories of 2084 known age individuals from 1992–2008 using an extension of the Cormack-Jolly-Seber model for open populations accounting for age-class specific variation in survival [16]. Error bars represent 95% confidence intervals.

**Fig 2:**
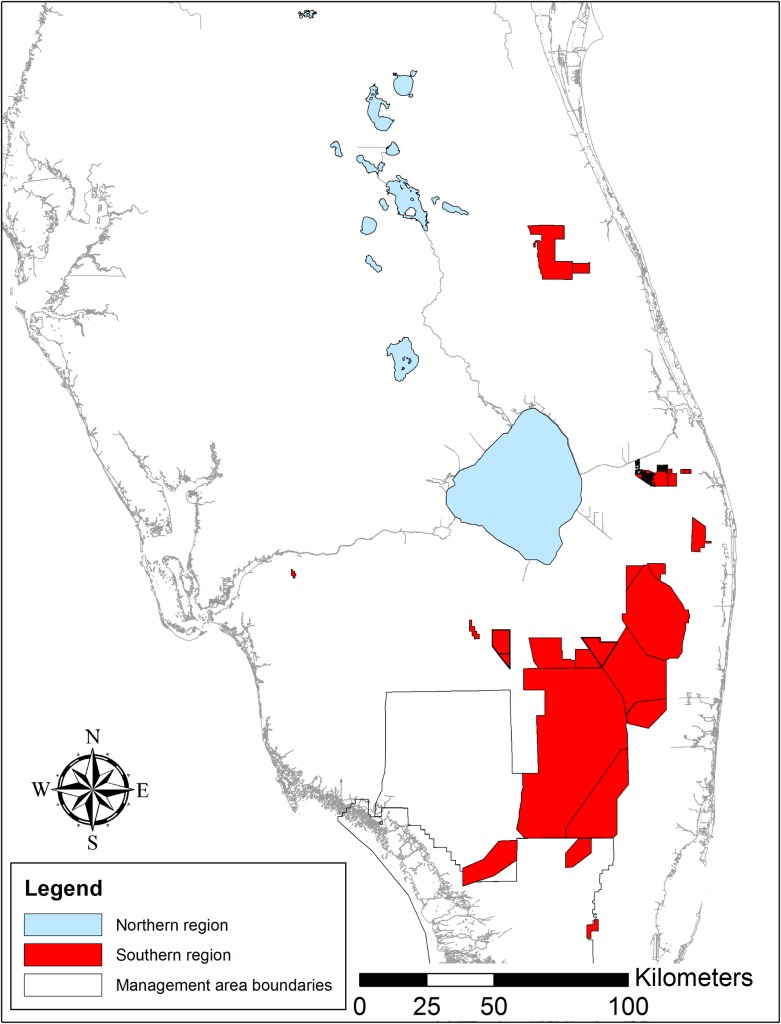
The primary wetlands located within the breeding range of the snail kite in Florida that were surveyed from 1997–2013. Wetlands that comprise the snail kite’s breeding range in Florida were previously classified into two regions (north and south) based on patterns of observed annual dispersal between wetlands [20]. All wetlands were systematically surveyed for banded and unbanded snail kites multiple (4–6) times during the peak of the snail kite breeding season (March 1^st^–June 30^th^) from 1997–2013.

Understanding both temporal and spatial variation in age structure is needed for the conservation of snail kites. From a temporal perspective, snail kites are long-lived birds that exhibit age-dependent vital rates, including senescent declines in adult survival beginning at age 13 [16] (Fig 1) and declines in breeding effort beginning at approximately age five [17]. In Florida, snail kites utilize a network of wetlands but tend to exhibit philopatry to their natal region [18] and relatively high site fidelity [19,20]. Recently, Reichert et al. [20] identified two regions of wetlands used by snail kites during the breeding season based on patterns of annual dispersal and movement, where the relative amount of observed movement was greater within versus between regions (see also [21]). One of these identified regions consists of wetlands in the northern half of the snail kite’s range (Fig 2), where breeding habitat is located primarily within or adjacent to lake littoral zones (herein referred to as the ‘Northern region’). Wetlands within the other identified region are located mostly south of Lake Okeechobee (Fig 2) and are typically dominated by shallow expansive, graminoid marshes (herein referred to as the ‘Southern region’) [22,23] (Fig 2). Recruitment of juveniles from the northern region has increased significantly since 2005, and has been substantially greater than juvenile recruitment from the southern region [24], where immigration has also been minimal [25]. Based on these lines of evidence, we made the following predictions. First, we predicted that the age distribution of snail kites in southern region has shifted over time towards a disproportionately high number of older birds compared to the age distribution of birds inhabiting the northern region. Second, we predicted that limited recruitment in the southern region has resulted in a regional decline in snail kite abundance.

## Materials and Methods

### Data collection

Beginning in 1976, juvenile snail kites were captured and banded just prior to fledging using individually-identifiable alpha-numeric colored leg bands [16]. Snail kites are a state and federally listed endangered species. Banding was conducted under US Geological Survey BBL Permit #23906 and USFWS permit #TE55292B-0. Since 1997, multiple band-resight surveys (4–6) have been conducted throughout the peak of the breeding season (March 1 to June 30) [26]. Sampling for each survey lasts 18–21 days and covers the entirety of the known breeding range of snail kites in Florida including all historical breeding sites [27] and wetlands where active nesting was encountered either by field technicians or reported by managers or the public throughout the breeding season (S1 Table). We conducted research on both private and public lands (see S1 Table for list of coordinates). We received explicit permission from landowners to conduct surveys on private lands (i.e. Devil’s Garden Bird Park, Hendry County, FL, and Shingle Marsh, Osceola County, FL). We also obtained required access permits to conduct research on public lands including Stormwater Treatment Areas 1,2,3,4,5, and 6 (South Florida Water Management District, Agreement #4600003005-A1), Arthur R. Marshall Loxahatchee National Wildlife Refuge (SUP B15-001), Everglades National Park (EVER-SCI-0062), and Big Cypress National Preserve (BICY-SCI-001). During each survey, the wetland locations of both banded and unbanded individuals are recorded. From 1997 through 2013, a total of 693 banded individuals of known age were re-sighted at least once as adults (≥1 years old) within the northern region and 649 individuals were re-sighted at least once as adults within the southern region (Fig 2). Data are available from the Dryad Digital Repository: http://dx.doi.org/10.5061/dryad.fg007. The study was approved and conducted under the guidelines of the University of Florida Institutional Animal Care and Use Committee (IACUC no. 201005469).

### Model description

We employed a superpopulation estimator for each region based on the open robust design multistate model (ORDMS) [28,29]. To evaluate the importance of accounting for spatial structure, we also conducted analyses at the scale of the entire geographic range of the species in Florida. The ORDMS includes modeling both within-season and between-season dynamics. However, in this analysis we were interested only in comparing trends between regions from independently calculated annual estimates of within-season abundance and the distribution of individuals within age classes (or age structure). Therefore, we used only the within-season component of the model, modeling each primary period (year) separately [30]. Using the ORDMS provides a convenient approach to assess both regional and age-related variation in abundance.

We implemented the ORDMS for separate years in Program MARK 7.2 [30,31] for which there are three parameters of interest. These parameters include: (i) the probability that an individual of state *s* moves into the study area at time *t*, ${pent}_{t}^{s},$ (ii) the probability that an individual of state *s* remains in the study area at time *t*, $\phi_{t}^{s}$, (iii) and the probability that the individual in state *s* is detected at time *t* given it is available $p_{t}^{s}$. We built an *a priori* model set for each year that included models with varying constraints on *pent* and *ϕ* (age class, region, time (i.e. survey), linear time effects, time-region interaction) (S2 Table and S3 Table). Models assumed that the probability of detecting an individual given it was present in the study area could vary between regions, among age classes, and/or survey periods.

### Age distribution

Models accounting for spatial structure (two regions) included six total states based on three age classes for each region, while range-wide analysis included only three age classes. Age classes were based on known variation in snail kite reproduction [7] and survival [16] and included subadults (1 year old), prime-aged adults (2–12 years old), and senescent adults (>12 years old). Because sampling occurred during the four-month breeding season, individuals remain in the same age class throughout all survey sessions within a year. To assess the proportion of individuals in each adult age class, we derived abundance estimates of the number of banded individuals in each age class (age-specific ‘superpopulation’) using Program MARK 7.2. Derived estimates were based on model-averaged parameter values for each year/region and range-wide populations (S2 Table and S3 Table). Importantly, our approach to assessing population age distribution makes the assumption that age distribution of banded birds is representative of the population’s age distribution across year/region scales (see S1 Fig for total counts of banded and unbanded snail kites).

### Superpopulation size

In addition to assessing changes in age structure, we were also interested in estimating regional breeding season abundance (including both banded and unbanded individuals). We first calculated the ‘superpopulation’ size of all banded adults $\left({\hat{M}}_{r}^{*}\right)$, or the total number of banded adults that were present and available in region, *r*, during at least one survey period within a breeding season, as:

$${\hat{M}}_{r}^{*}=\sum_{s=1}^{3}\frac{m_{r}^{s}}{{\hat{p^{*}}}_{r}^{s}}$$

where $m_{r}^{s}$ is the number of banded individuals observed in adult age class, *s*, within region, *r*, and ${\hat{p^{*}}}_{r}^{s}$ is the probability that an individual of age, *s*, in region *r*, was re-sighted at least once [28,30]. We estimated the variance of ${\hat{M}}_{r}^{*}$ via the delta method using the variance-covariance matrix for the estimates of banded adults in each age class, $\frac{m_{r}^{s}}{{\hat{p^{*}}}_{r}^{s}}$ provided by Program MARK.

In a traditional capture-mark-recapture analysis, new individuals are captured and banded during each survey session. In such cases, ${\hat{M}}_{r}^{*}$ would include all individuals (banded and unbanded) that were alive and available for capture during at least one survey session (i.e. superpopulation size). Because snail kites are banded at the nest as fledglings and we were only interested in the superpopulation size of adults, the superpopulation size for both banded and unbanded individuals (${\hat{N}}_{r}^{SUPER}$) was calculated by adjusting ${\hat{M}}_{r}^{*}$ by the proportion of individuals observed in region *r* across all six surveys which were banded, as follows:

$${\hat{N}}_{r}^{SUPER}=\frac{{\hat{M}}_{r}^{*}}{\frac{m_{r}}{m_{r}+u_{r}}}$$

where *m*_*r*_ and *u*_*r*_ are the total number of banded and unbanded individuals re-sighted in region *r* during all surveys, respectively (S1 Fig). We calculated the variance of ${\hat{N}}_{r}^{SUPER}$ using the estimated variance of ${\hat{M}}_{r}^{*}$ (described above) and the delta method in R [32], using package ‘msm’ [33] assuming that counts of snail kites were Poisson distributed with a mean and variance equal to the observed counts (*m*_*r*_ and *u*_*r*_).

We used generalized additive models (GAM) to assess trends in abundance and to test for significant differences in regional trends. To account for uncertainty in estimates of superpopulation size, we weighted GAM by the inverse of the standard deviation of the annual estimates using the R-package ‘mgcv’ (version 1.8–10) [34]. We then used an F-test to compare GAM with and without an additional smoother spline (‘region x s(year)’ interaction term) and assessed significance at the α = 0.05 level [34,35]. We used ‘finite difference’ methods on the GAM predictions to identify periods of significant increase and decrease for each region and at the range-wide scale [34].

## Results

### Models with spatial structure

For all years, the model best supported by the data (based on AIC model selection) assumed that the probability of snail kites entering the study area within a given year (primary period) (*pent*) varied by survey session (Table 1). In 10 of 17 years, there were significant differences in the survey-specific estimates of *pent* and in all of these years a significant decline occurred after the first survey (S2 Fig).

**Table 1:** Summary of best supported ORDMS models used to estimate snail kite population size and age structure from 1997–2013, that incorporate regional variation (a) versus models for the entire breeding range (b). **Bold** indicates significant effect based on non-overlapping 95%CI. See Supporting Information for associated point estimates and 95%CI of those parameters (S2 Fig and S3 Fig), and more details on all models considered (S2 Table and S3 Table).

	Year	*pent*	*ϕ*	*p*	AICc Weights	Num. Par
(a)	** **	** **	** **	** **	** **	** **
	1997	survey (categorical)	survey (categorical)	region	0.41	14
	1998	survey (linear)	age	region*survey (categorical)	0.97	19
	1999	**survey (linear)**	age	region*survey (categorical)	0.90	19
	2000	**survey (linear)**	constant	region	0.45	7
	2001	survey (linear)	age	region*survey (categorical)	0.97	19
	2002	survey (linear)	constant	region	0.87	7
	2003	survey (linear)	age	age	0.46	10
	2004	survey (linear)	age	region*survey (categorical)	0.88	19
	2005	**survey (linear)**	age	region*survey (categorical)	1.00	19
	2006	**survey (linear)**	age	region*survey (categorical)	1.00	19
	2007	**survey (linear)**	age	region*survey (categorical)	0.95	19
	2008	survey (linear)	constant	**region**	0.52	7
	2009	**survey (linear)**+age	age*survey (categorical)	**region**	0.61	16
	2010	**survey (linear)**	age	region*survey (categorical)	0.42	19
	2011	**survey (linear)**	age	region*survey (categorical)	0.77	19
	2012	**survey (categorical)**	**age**	**region**	0.84	12
	2013	**survey (categorical)**	age	**age**	1.00	13
(b)						
	1997	survey (categorical)	constant	survey (categorical)	0.62	14
	1998	**survey (linear)**	age	constant	0.70	8
	1999	**survey (linear)**	constant	constant	0.47	6
	2000	**survey (linear)**	constant	constant	0.61	6
	2001	survey (categorical)	constant	constant	0.65	9
	2002	**survey (linear)**	constant	constant	0.44	6
	2003	survey (linear)	constant	constant	0.40	6
	2004	survey (linear)	age	constant	0.61	6
	2005	survey (linear)	constant	constant	0.27	6
	2006	**survey (categorical)**	constant	constant	0.54	9
	2007	**survey (linear)**	constant	constant	0.73	6
	2008	survey (linear)	constant	constant	0.47	6
	2009	**survey (linear)**	**age**	constant	0.53	11
	2010	**survey (categorical)**	**age**	constant	0.45	11
	2011	**survey (linear)**	**age**	constant	0.79	11
	2012	**survey (linear)**	**age**	constant	0.91	11
	2013	**survey (linear)**	**age**	constant	0.98	11

*Model notation*: “pent” = survey-specific probability of a snail kite entering the study area, “*ϕ*” = survey-specific probability that a snail kite remained in the study area, “p” = probability that a snail kite was detected in the study area given it was available, “constant” = model parameter was assumed to be constant, “survey (categorical)” = model parameter varied among surveys, “survey (linear)” = model parameter varied as a linear function of time (by survey), “age” = model parameter varied between age class (0–1 years, 2–12 years, 13+ years), and “region” = model parameter varied for individuals in different regions. *Examples of model interpretation*: model for 1997 (a) assumes the probability that an individual located outside the surveyed wetlands moved into the study area (pent) and the probability that an individual remained in the study area (***ϕ***) differed between survey periods, while the probability of observing an individual that was present within the study area (p) differed between regions. Model for 1998 (a) assumes that the probability of an individual entering the study area changed linearly from the first to the last survey (allowed to increase or decrease), the probability that an individual stayed within the study area depends on the age class of the individual, and the probability of observing an individual present in the study area varied between survey periods and regions (i.e. interactive effect).

In 13 of 17 years, the best supported model included age effects on the probability of a snail kite remaining in the study area between consecutive surveys, *ϕ* (Table 1). However, estimates for subadults varied considerably and 95% confidence intervals overlapped such that we only found significant differences in age-dependent estimates for 2012 (Table 1) during which estimates were significantly lower for individuals in the senescent age class (S2 Fig).

In most years (15 of 17), the best supported model included regional effects on detection probability (Table 1). For eight of those years, estimates of detection probability were significantly different between regions with higher detection probability of individuals in the northern versus the southern region (S2 Fig). In five of the years, estimated detection varied significantly between regions and survey sessions later in the breeding season when again, individuals in the northern region were detected at higher rates compared to those in the southern region (S2 Fig). For two of the 17 study years, models assuming age-dependent variation in detection were most supported by the data (Table 1). In 2013, detection probability varied significantly among age classes with lower detection of senescent compared to prime-aged adults (S2 Fig).

**Fig 3:**
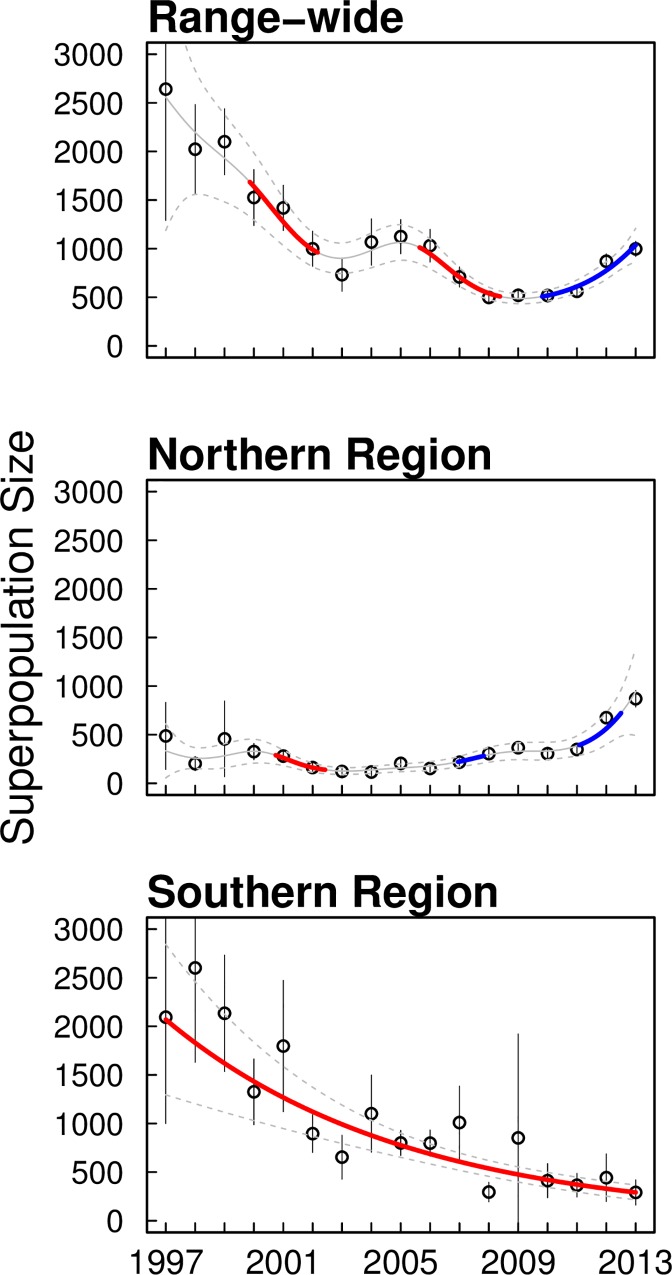
Superpopulation size of adult snail kites at the regional and range-wide scales. Derived point estimates of superpopulation size (⦁) with 95% CI’s of snail kites during the breeding season (March1-June 30^th^) in northern region, southern region, and over the entire breeding range. Estimates were derived based on model-averaged parameter values using extensions of the open robust design multistate model. Solid and dashed grey lines represent predicted mean and 95% CI’s from GAM used to assess trends in estimates of superpopulation size. Blue lines indicate periods of significant increase and red lines indicate periods of significant decrease in population size, both identified using methods of ‘finite differences’.

As predicted, temporal dynamics in population size were different between northern and southern regions of the snail kite’s range in Florida (Fig 3). Trends in abundance differed significantly between regions (F = 16.498, p-value<0.001). The northern region experienced a significant decrease from 2001 to 2002, then significantly increased from 2007 to 2008 and again from 2011–2013 (Fig 3). Estimated abundance of snail kites in the northern region increased roughly four-fold from 2007 (217, 95% CI = 181 to 253) to 2013 (870, 95% CI = 786 to 954) (Fig 3). In contrast, the southern region declined significantly throughout the study period (Fig 3) during which the estimated abundance declined dramatically from its highest estimate of 2,601 (95% CI = 1630 to 3572) in 1998 to 291 (95% CI = 163 to 420) individuals in 2013 (Fig 3).

**Fig 4:**
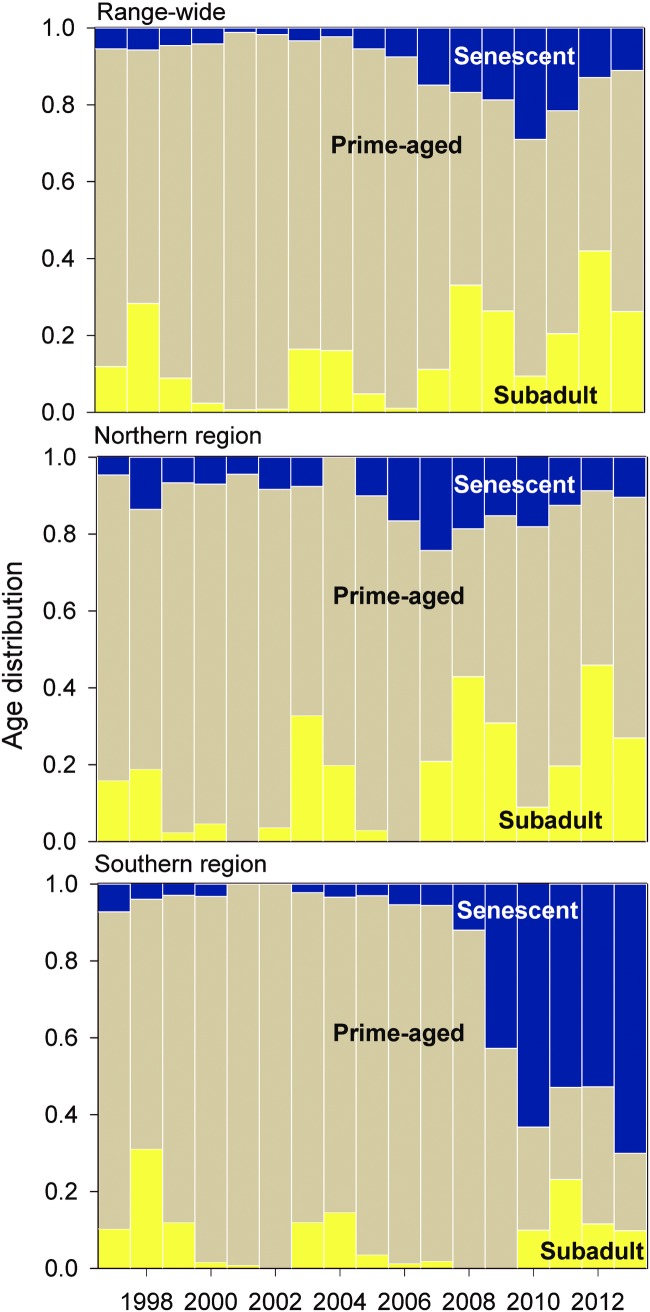
Age distribution of snail kites at the regional and range-wide scales. Estimated proportions of adult snail kites across three biologically-relevant age classes (0–1 years, 1–12 years, and 13+ years) in the northern region, southern region, and over the entire breeding range. Proportions of individuals in each age class were calculated using model-averaged estimates for the superpopulation size of banded individuals in each age class.

The two regions also had distinctly different age distributions over time (Fig 4). Since 2005, the southern region has had a steady and significant increase in the proportion of senescent individuals (13 years of age and older), such that in 2013, senescent adults comprised more than two-thirds (70.03%, 95% CI = 65.8 to 74.2) of snail kites in the region (Fig 4), as compared to only 10.5% (95% CI = 8.4 to 12.6) in the northern region. The greatest increase in the proportion of senescent adults in the south occurred from 2008 to 2009, during which the proportion of one year-old subadults was estimated to be zero. After 2009, the proportion of subadults increased, yet prime-aged adults in the south remained relatively low compared to pre-2009 estimates. Proportions of subadults in the northern region generally increased over the study period (Fig 4).

### Range-wide models

Results of modeling within-breeding season dynamics (*pent* and *ϕ*) at the range-wide scale were similar to the results from ORDMS with spatial structure. For all years, the best supported models included survey-dependent probabilities of snail kites entering the study area, *pent* (Table 1). In 11 years estimates of *pent* differed significantly between surveys and decreased during the breeding season (S3 Fig). The best supported models in seven different years included age-dependent effects on the probabilities of individuals remaining in the study area (Table 1). In five of these years, estimates were significantly lower for individuals in the senescent age class (S3 Fig). Unlike ORDMS with spatial structure, the best supported models in 16 of 17 years assumed detection probability was constant across surveys (Table 1). Age-dependent variation in detection probability was included in the best supported model for 1997, but estimates were not significantly different (Table 1).

Using pooled data from northern and southern subpopulations, we estimated abundance and age structure at the range-wide scale and compared it to estimates and trends from analyses that accounted for spatial population structure. Using the method of ‘finite differences’ on the predictions of GAM, we found that range-wide snail kite abundance declined significantly from 2000–2002 and again from 2006–2008 (Fig 3). Similar to the northern region, population size then increased significantly from 2010 to 2013 (Fig 3). From 1997 to 2013, superpopulation size ranged from an estimated high of 2,641 (95% CI = 1292 to 3990) in 1997 to a low of 498 (95% CI = 436 to 560) in 2013. Changes in age structure over time closely reflect that of the southern region until 2006. After approximately 2006, estimated range-wide age structure more closely reflect the age distribution in the northern region, as evidenced by the proportion of senescent individuals (Fig 4).

## Discussion

Aging effects on populations are of conservation concern because reduced proportions of younger individuals can alter population growth rates [36], potentially increasing the risk of short-term local extinction [10]. We identified regional differences in age structure and temporal trends in local abundance of a highly endangered population of snail kites in Florida. Specifically, there has been a significant population decline and shift towards a higher proportion of senescent adults in south Florida, a region comprised of wetlands historically considered to be critical habitat [37]. Our findings are especially alarming for snail kites in the region given that actuarial senescence occurs [16] (Fig 1). Unless local juvenile recruitment or immigration increases, snail kites in south Florida could experience continued population decline in the near-term and a substantial lag period before recovery, even if recruitment does increase [10,11].

### Potential drivers of variation in snail kite age structure and abundance

Changes in habitat quality can influence age structure and abundance through effects on local demography (survival and reproduction), dispersal, or a combination of both. Habitat quality can vary at local and large scales over time. Throughout the snail kite’s breeding range in Florida, large-scale environmental change may have range-wide (annual) effects on demography which could lead to synchronized changes in age structure between regions. However, we identified significant spatial variation in age structure and abundance and did not find evidence that proportions of age classes were correlated between regions (e.g., subadults, r = 0.167, P = 0.522, *see* S4 Fig). In the southern region of the snail kite’s range, several changes in local habitat quality have occurred, including changes in drought frequency, vegetation structure [38], and prey abundance [39]. These changes may have limited regional population growth [7] and altered local age structure.

Perhaps the most dramatic decline in the quality of snail kite habitat occurs during periods of drought which severely limit the availability of apple snails (*Pomacea* spp.) to foraging snail kites [40,41]. Northern lacustrine wetlands are less susceptible to complete desiccation during drought, which could explain regional variation observed in this study. Kites can escape the potential impacts of regional droughts by moving to unaffected wetlands [42–45], but the risk of mortality increases as a function of the spatial extent and duration of drought [43,45]. The negative impacts of drought on local snail kite survival [16,45,46] and breeding effort [47] may help, in part, to explain the reduced proportions of younger individuals and observed declines in snail kite abundance in the southern region. However, even in relatively wet years reproduction and juvenile recruitment has remained low in the southern region [48]. This suggests that other key processes such as long-term changes in the hydrologic regime have also negatively impacted snail kite foraging habitat (e.g. [38,49–51]) and prey abundance [52] and likely contributed to observed changes in local demography [7].

In addition to a decline in habitat quality in the south, increases in local habitat quality have occurred simultaneously in the north, which have likely exacerbated regional differences in demography. Recent increases in local reproduction and population growth in the northern region have been largely attributed to the invasion of a non-native species of apple snail (*Pomacea maculata*) [24]. Snail kite movements to invaded wetlands have also increased; however, the probability of a kite dispersing was relatively small compared to the probability of remaining in the south [24]. We found that a disproportionately high number of older individuals used wetlands in the southern region despite changes in habitat quality. While emigration of juvenile and prime-aged adults out of the southern region may have contributed at least minimally to the observed population declines in the south, reduced local reproduction and juvenile survival have likely played a larger role in the observed changes in snail kite age structure and abundance. For example, from standardized monitoring from 1997 through 2004, 1,215 nests were monitored and 793 were young banded in this region. Yet from 2005 through 2013, only 386 nests were found and 125 young were banded [24]. Furthermore, juvenile survival in the southern region was significantly lower (~36%) from 2005 to 2013 compared to the period of 1992 to 2004 [24]. This decline in reproductive effort and juvenile survival likely limited local recruitment and population growth in the region [7].

### Population estimation with and without structure

**Fig 5:**
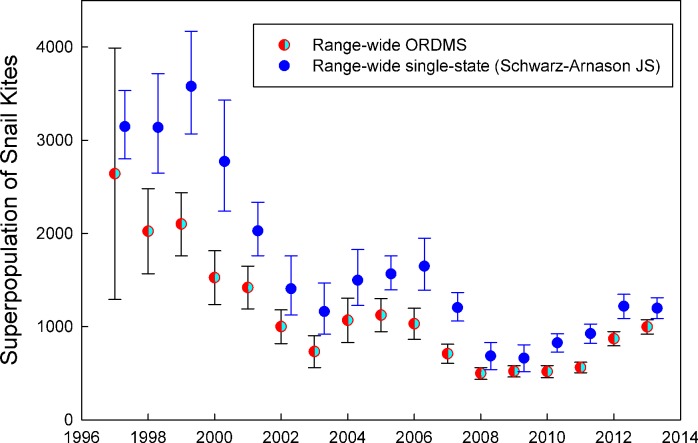
Comparison of estimates with 95% CIs of range-wide superpopulation size of snail kites during the breeding season in Florida using single-state and open robust design multistate models.

We developed a modeling framework using the ORDMS that accounted for spatial structure and compared the results to models that ignored spatial structure by pooling data across the entire range of the study population. While range-wide abundance estimators using the ORDMS can account for several sources of variation, including temporal variability in movement rates in and out of the study area, they do not typically deal with spatial variation in abundance and age structure. By incorporating spatial population structure, we were able to identify habitat-specific (regional) trends in demography and a multi-year shift in snail kite abundance between regions. These patterns would have been missed if age structure was only assessed at the range-wide scale (Figs 3 and 4). Estimates of range-wide abundance were lower using the multistate approach compared to estimates from a single-state superpopulation estimator reported previously by Dreitz et al. [27] and Martin et al. [53]. However, temporal trends were very similar between estimators (Fig 5). Differences between estimates using the two approaches may be an effect of pooling parameters for the single state estimator, which ignores age-related and spatial sources of heterogeneity in detection. Further work is needed to investigate the differences between estimates using the two approaches.

Inferences from our analysis rely on the assumption of representativeness, in which the age structure of banded birds over time is representative of the age structure of the entire (sub)population. However, this assumption is likely reasonable in our case for two main reasons. First, banding of nestlings began well before the onset of our analysis (>5 years). Second, banding effort, which involves attempting to band all nestlings at every known nest through the snail kite’s range, has remained relatively consistent across both regions over time.

### Conservation implications

Population viability analysis is an important tool in conservation biology [5,6]. Traditional approaches often assume long-term asymptotic growth and a stable-age distribution [2]. We present an empirical example of how age structure varies through time and across the geographic range of an endangered population. Such changes in age structure can give rise to short-term transient population dynamics that can influence realized growth rates [54–56] and alter extinction probability [57]. Our results emphasize that the assumption of stable age distribution may not be valid, especially for small populations whose vital rates are strongly influenced by local habitat conditions and exist in spatially and temporally heterogeneous environments (see also [58]).

Age structure is a common characteristic of most animal populations and it has consequences for population dynamics [59,60]. For snail kites, our results and related findings [24] suggest that local habitat quality is likely driving age structure dynamics more than dispersal. Because these two issues can have very different implications for conservation practice, we argue that further work on age structure needs to isolate the factors driving age structure dynamics. By estimating variation in age structure, we revealed a hidden potential issue for the conservation of this endangered species—that the snail kite population in the southern region will likely experience continued suppressed population growth in the near term. Information on changes in age structure, like that provided here, might provide ‘early warning signals’ for population decline in a variety of populations that are limited by habitat quality.

These results show that despite the fact that across their range, snail kites have recently increased in population size (Fig 3, [24]), regional differences are considerable in terms of population abundance and age structure. Population assessments need to include regional trends rather than relying solely on trends across the entire population. Such regional variation will help identify factors limiting the recovery of snail kites in Florida.

Currently, greater recruitment of young birds is needed in south Florida—a region of vast amounts of potential habitat. This will require strategies to improve nesting success (e.g., water management) and foster natal and breeding dispersal to the southern region. While a small pool of potential immigrants may exist that have remained outside of the study area in recent years, these individuals are likely not sufficient for the recovery of the population in the southern region. Better understanding how management can increase recruitment in palustrine wetlands and how to reduce effects of key factors, such as droughts is needed. While we have an increasing understanding of how environmental variation can impact reproduction (e.g.,[39]), integrated approaches with managers is needed to better interpret the efficacy of management options.

The Everglades is the largest restoration effort in the world. Currently, there is a great opportunity for improving habitat quality in the southern region via the restoration process. The snail kite is a key indicator for Everglades restoration [61]. Recommendations for managing hydrology to improve snail kite breeding habitat have been incorporated into larger multispecies planning efforts (e.g. [62]). The trends we revealed here, however, support recent findings that, to date, attempts to restore the hydrology of the historical Everglades have not been sufficient [63] and highlights the urgency for restoration. As restoration proceeds, we need to better understand how to manage hydrology for the needs of wildlife given a multitude of constraints, including human population growth and predicted increases in drought frequency due to climate change [64].

## Supporting Information

S1 Fig Total counts of banded and unbanded snail kites across all breeding season surveys for northern and southern regions (n = 4–6) from 1997–2013. (PDF)

S2 Fig Estimated probabilities of an individual snail kite entering (*pent*), remaining (*ϕ*), and being detected (*p*) in the study area from an open robust design multistate model with spatial structure (two regions). **Error bars represent 95% confidence intervals.** Estimates are from models best supported by the data with significant effects of survey period (linear and categorical), age class (categorical), region (categorical), or region x survey period interactions. Models fit to band-resight data collected over multiple standardized range-wide surveys conducted at the peak of the snail kite breeding season (March 1^st^–June 30^th^).
(PDF)

S3 Fig Estimated probabilities of an individual snail kite entering (*pent*), remaining (*ϕ*), and being detected (*p*) in the study area from an open robust design multistate model at the range-wide scale. **Error bars represent 95% confidence intervals.** Estimates are from models best supported by the data with significant effects of survey period (linear and categorical), age class (categorical), region (categorical), or region x survey period interactions. Models fit to band-resight data collected over multiple standardized range-wide surveys conducted at the peak of the snail kite breeding season (March 1^st^–June 30^th^).
(PDF)

S4 Fig Scatterplot of the proportions of subadults in northern and southern regions. Subadult proportions were not correlated (r = 0.167, p = 0.522).
(PDF)

S1 Table Summary of surveyed wetlands including access information (public versus private), access permit numbers where applicable (public lands), and site coordinates (WGS 1984 UTM Zone 17N). (PDF)

S2 Table Model results used to obtain model-averaged parameter values for derived estimates of snail kite superpopulation size and age structure during the breeding season (March 1 –June 30) from 1997 through 2013 in Florida using an open robust design multi-state model with spatial structure (two regions). Model notation: “pent” = survey-specific probability of a snail kite entering the study area, “phi” = survey-specific probability that a snail kite remained in the study area, “p” = probability that a snail kite was detected in the study area given it was available, “.” = model parameter was assumed to be constant, “time” = model parameter varied among surveys, “Time” = model parameter varied as a linear function of time (by survey), “age” = model parameter varied between age class (0–1 years, 2–12 years, 13+ years), and “region” = model parameter varied for individuals in different regions.
(PDF)

S3 Table Model results used to obtain model-averaged parameter values for derived estimates of snail kite superpopulation size and age structure during the breeding season (March 1 –June 30) from 1997 through 2013 at the range-wide scale using an open robust design multi-state model. Model notation: “pent” = survey-specific probability of a snail kite entering the study area, “phi” = survey-specific probability that a snail kite remained in the study area, “p” = probability that a snail kite was detected in the study area given it was available, “.” = model parameter was assumed to be constant, “time” = model parameter varied among surveys, “Time” = model parameter varied as a linear function of time (by survey), and “age” = model parameter varied between age class (0–1 years, 2–12 years, 13+ years).
(PDF)
