# Supplementary figures and images for: Spatio-Temporal Variation in Age Structure and Abundance of the Endangered Snail Kite: Pooling across Regions Masks a Declining and Aging Population
Source: PLoS One. 2016 Sep 28;11(9):e0162690. doi: 10.1371/journal.pone.0162690 (PMC5040393; doi:10.1371/journal.pone.0162690)

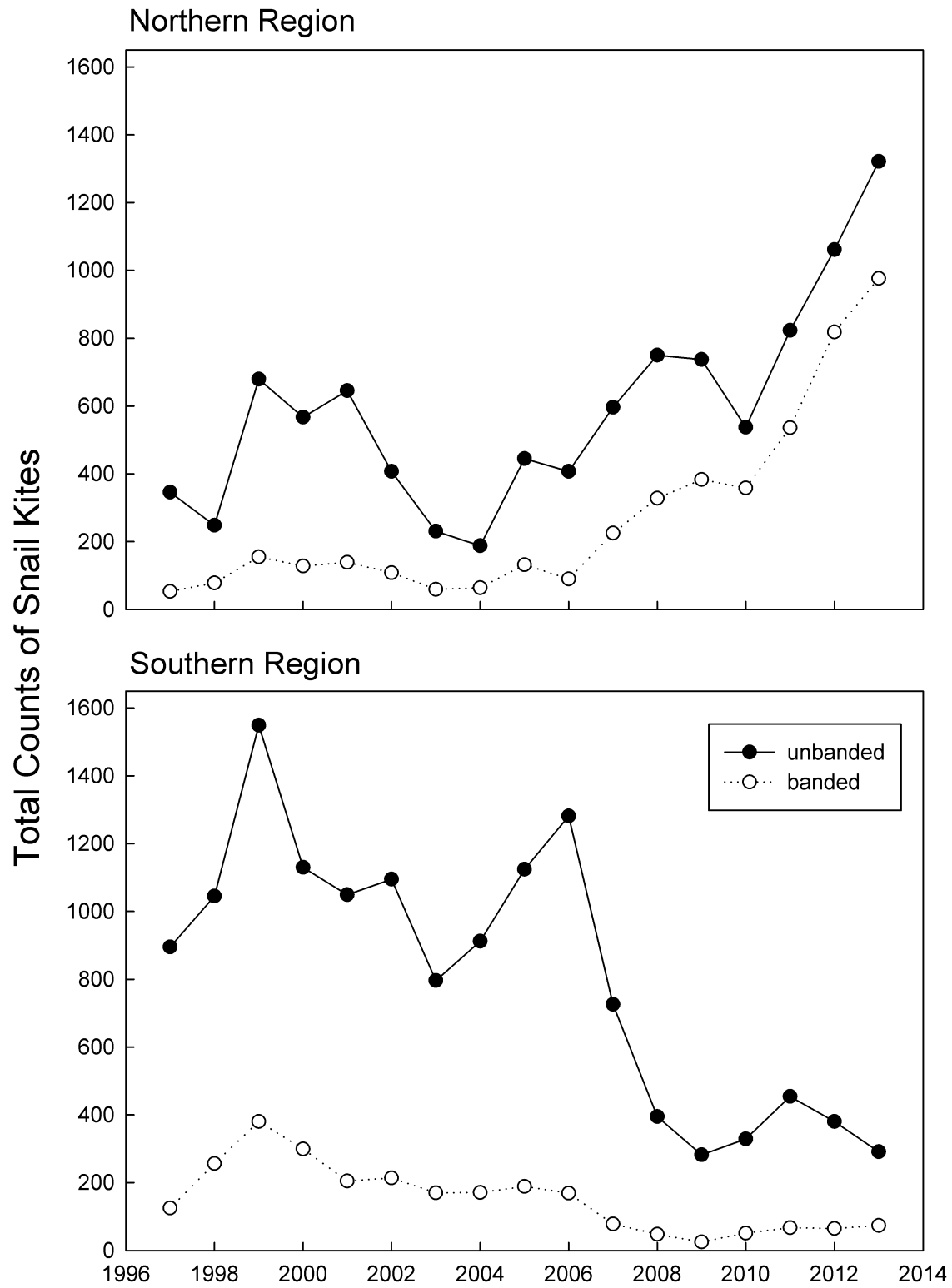

Supplement: S1 Fig — (PDF) [file pone.0162690.s001.PDF]

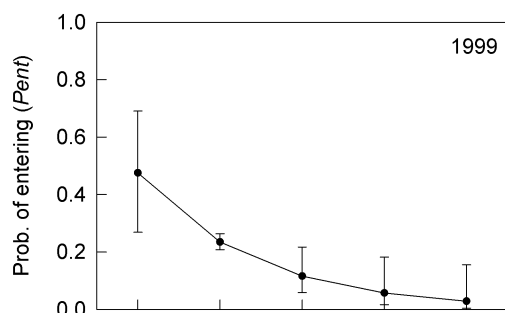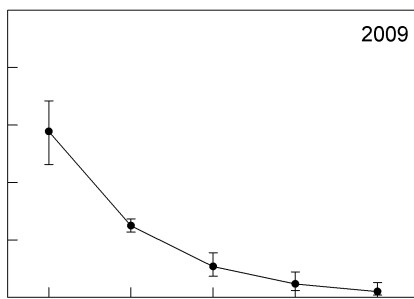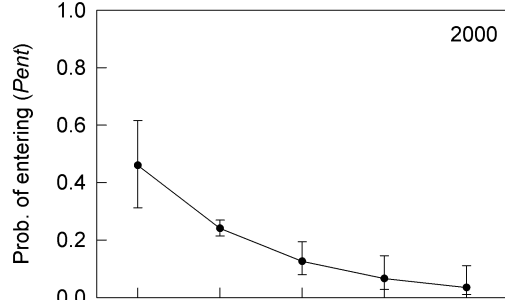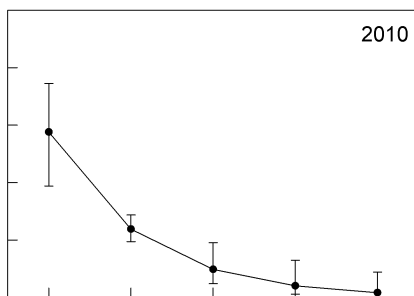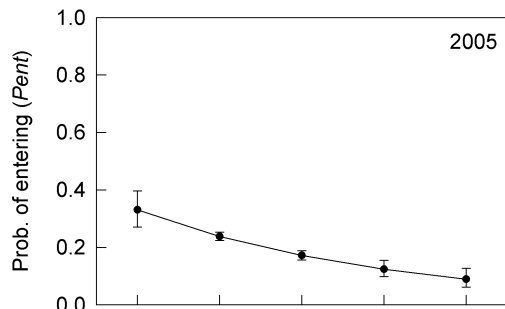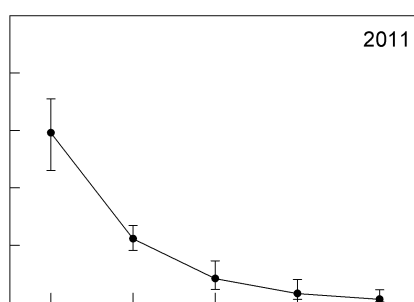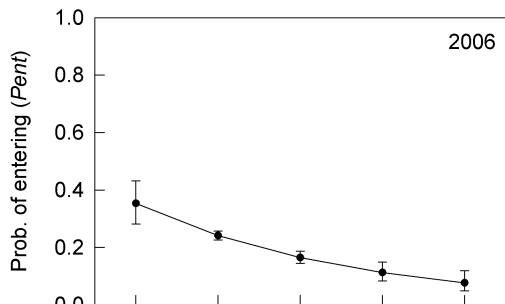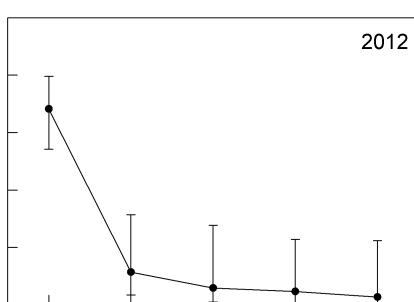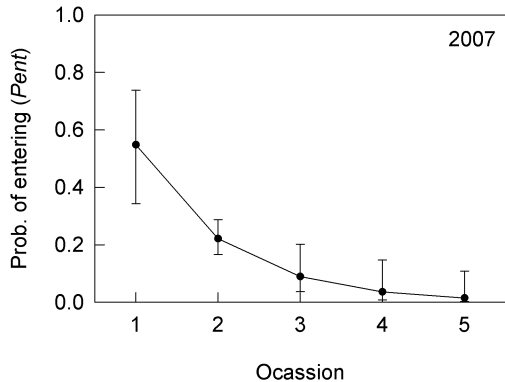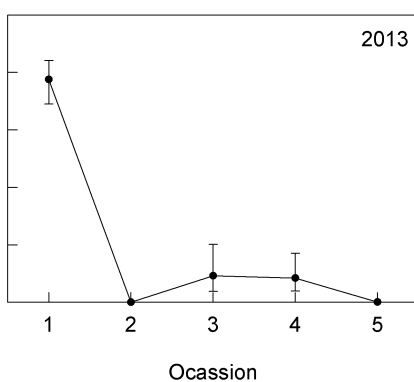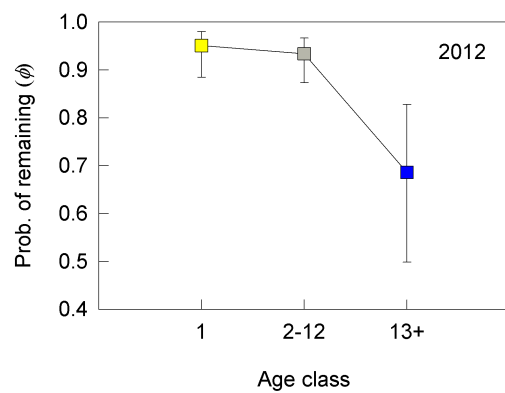

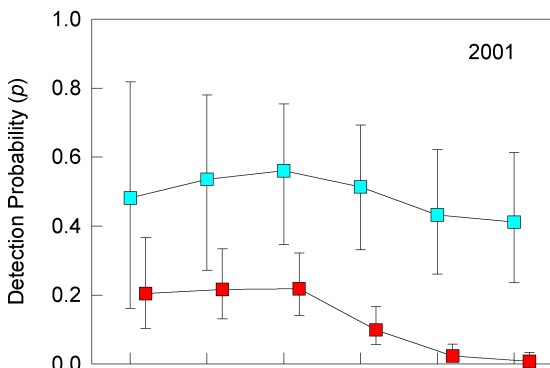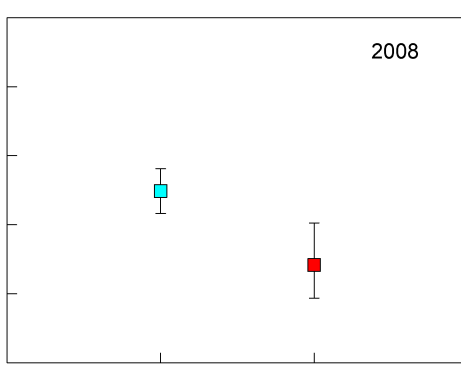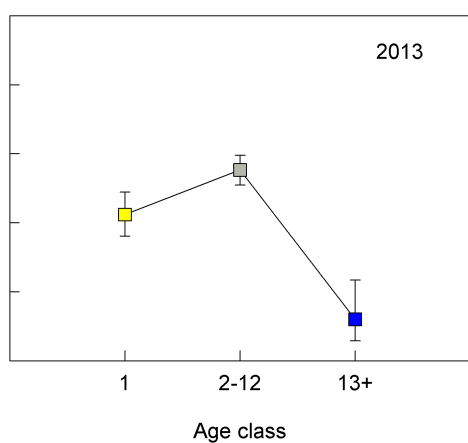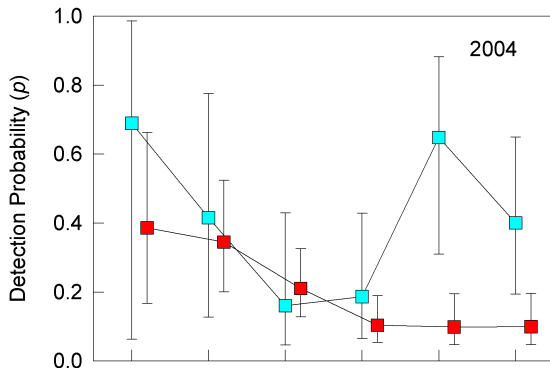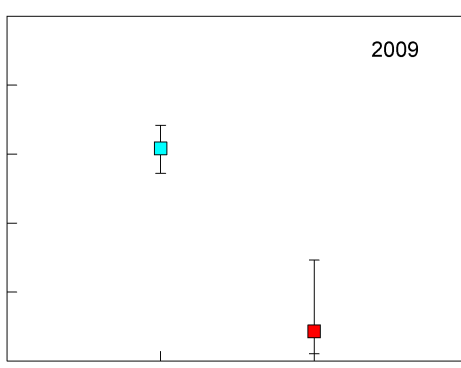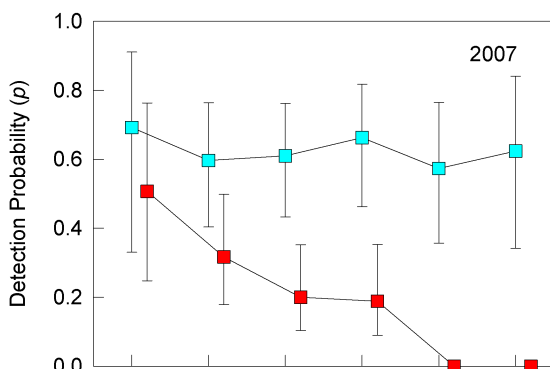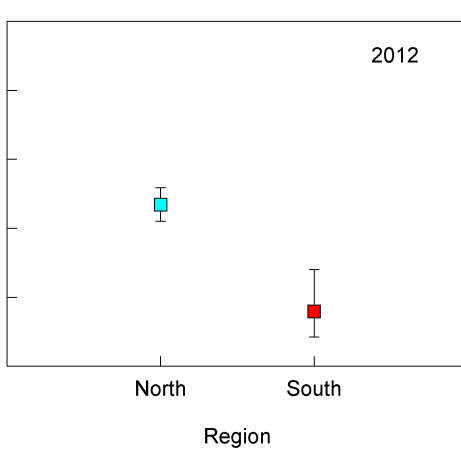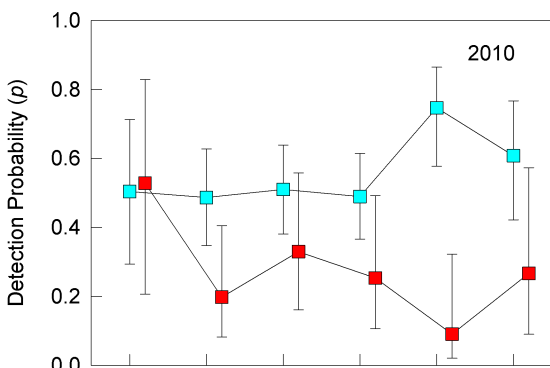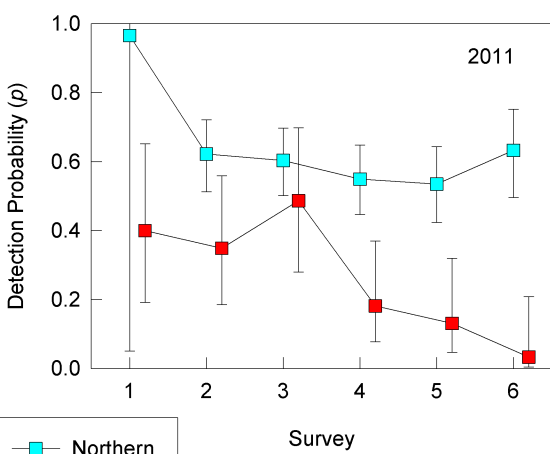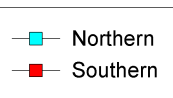

Supplement: S2 Fig — Error bars represent 95% confidence intervals. Estimates are from models best supported by the data with significant effects of survey period (linear and categorical), age class (categorical), region (categorical), or region x survey period interactions. Models fit to band-resight data collected over multiple standardized range-wide surveys conducted at the peak of the snail kite breeding season (March 1st–June 30th). (PDF) [file pone.0162690.s002.PDF]

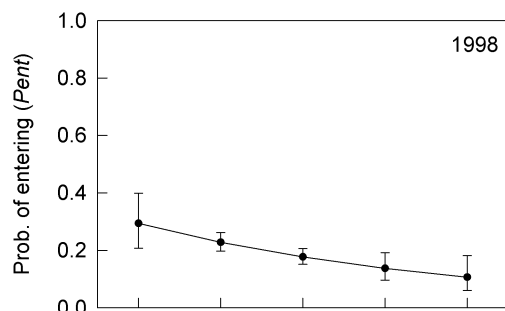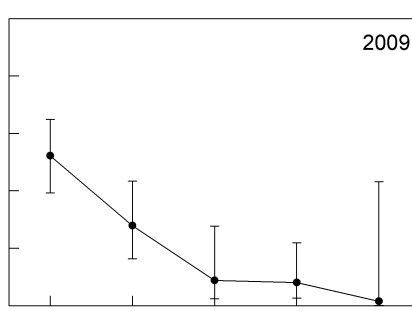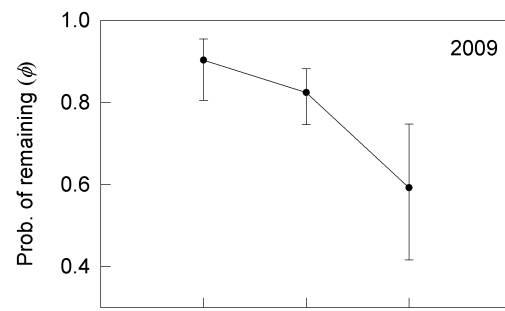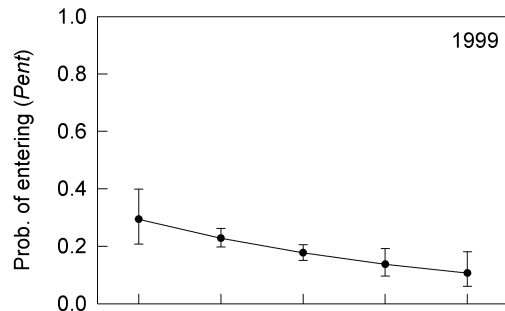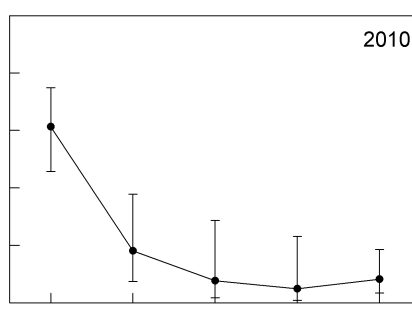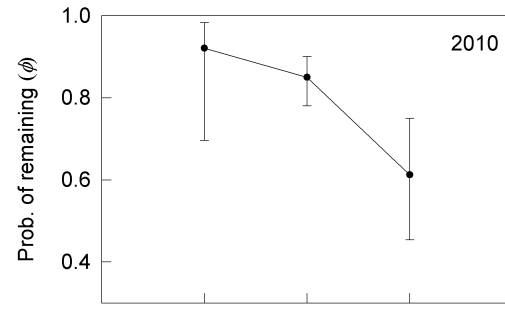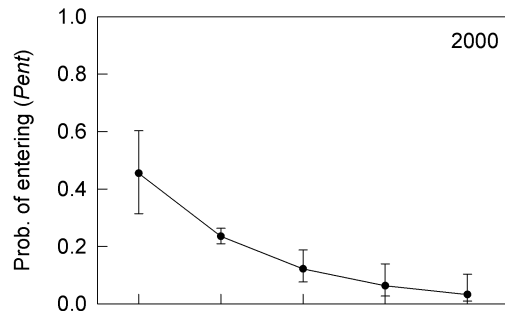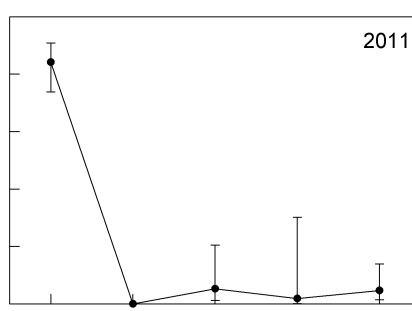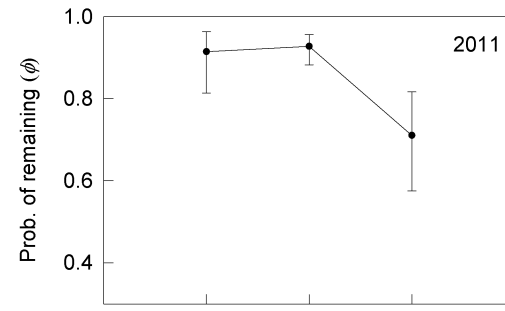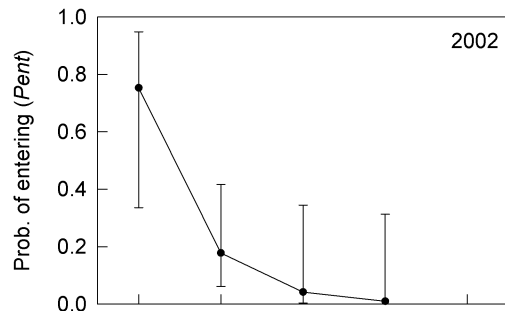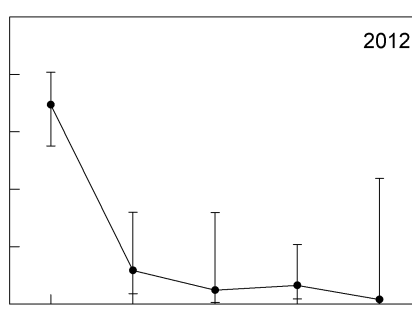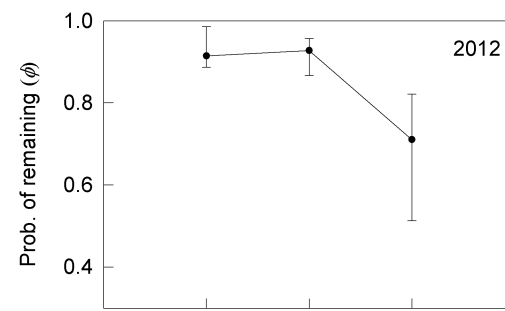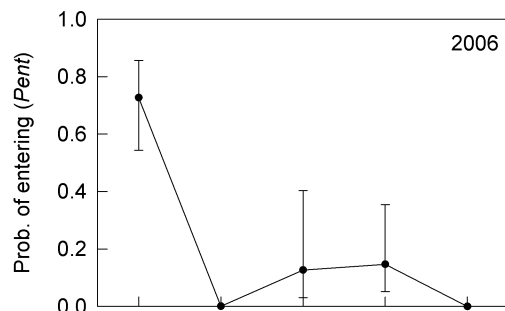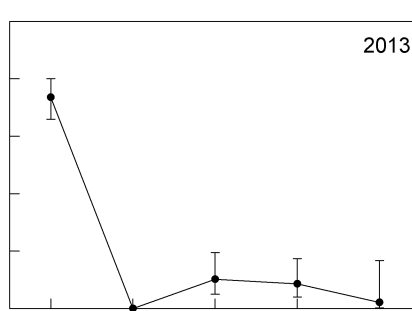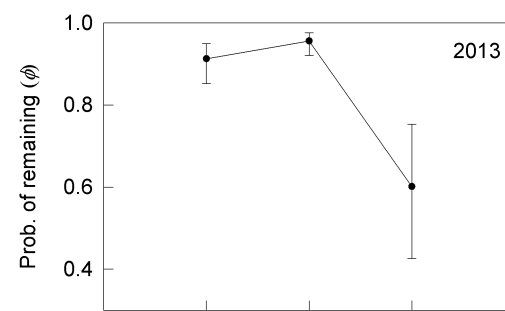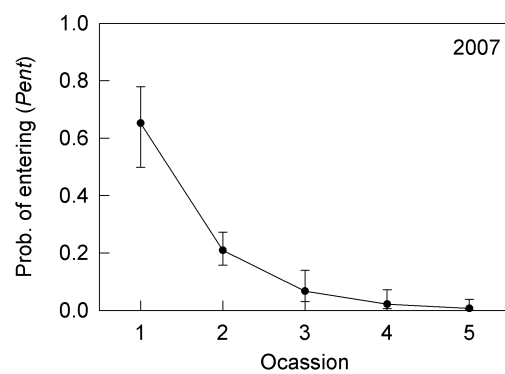

Supplement: S3 Fig — Error bars represent 95% confidence intervals. Estimates are from models best supported by the data with significant effects of survey period (linear and categorical), age class (categorical), region (categorical), or region x survey period interactions. Models fit to band-resight data collected over multiple standardized range-wide surveys conducted at the peak of the snail kite breeding season (March 1st–June 30th). (PDF) [file pone.0162690.s003.PDF]

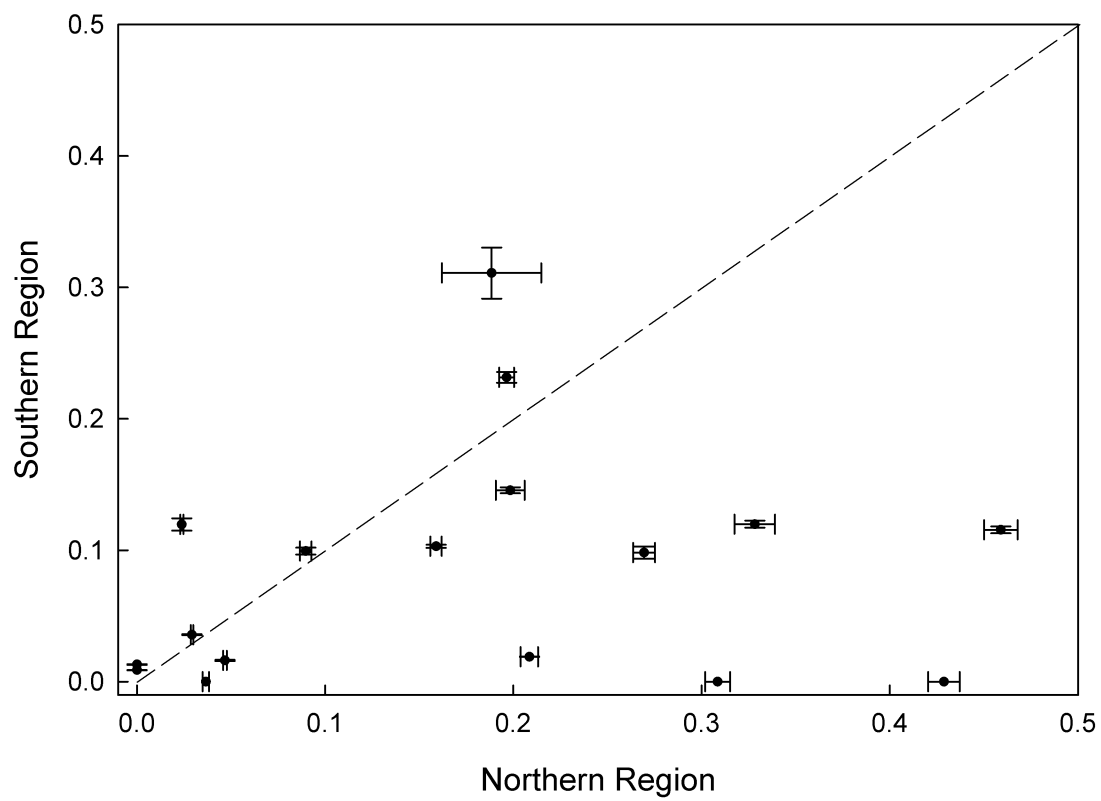

Supplement: S4 Fig — Subadult proportions were not correlated (r = 0.167, p = 0.522). (PDF) [file pone.0162690.s004.PDF]
