# Supplementary material for: Spatio-Temporal Variation in Age Structure and Abundance of the Endangered Snail Kite: Pooling across Regions Masks a Declining and Aging Population
Source: PLoS One. 2016 Sep 28;11(9):e0162690. doi: 10.1371/journal.pone.0162690 (PMC5040393; doi:10.1371/journal.pone.0162690)
Supplement: S1 Table — (PDF) [file pone.0162690.s005.pdf]

Site name	Access	Permit	X Coordinate	Y Coordinate
Arthur R. Marshall Loxahatchee National Wildlife Refuge	public	SUP B15-001	566043	2931840
Big Cypress National Preserve	public	BICY-2016-SCI-0001	495505	2849390
Blue Cypress Water Management Area (and adjacent wetlands)	public		536632	3061140
City of West Palm Beach Grassy Waters Preserve	public		582883	2962720
Cypress Lake	public		468212	3105990
Devils Garden Bird Park	private		497670	2925290
East Lake Tohopekaliga	public		472324	3130400
Everglades National Park	public	EVER-2014-SCI-0062	531302	2840110
Frog Pond/Transition Lands/L31	public		543938	2819856
Harns Marsh, Lehigh Acres	public		431024	2948020
Holey Land Wildlife Management Area	public		526531	2917990
Hungryland Wildlife and Evironmental Area	public		569598	2983060
Johns Lake	public		436482	3156301
Lake Arbuckle	public		460908	3063198
Lake Hatchineha	public		459656	3100220
Lake Istokpoga	public		472176	3028470
Lake Jackson	public		484077	3087110
Lake Kissimmee	public		473993	3087280
Lake Marian	public		491042	3083720
Lake Marion	public		447430	3106116
Lake Okeechobee	public		501592	2979150
Lake Runnymede	public		475011	3125890
Lake Tarpon	public		330797	3111280
Lake Tohopekaliga	public		462377	3120320
Lake Weohyakapka	public		459193	3077472
Shingle Marsh	private		459376	3127320
Stormwater Treatment Area 1 (South Florida Water Management District)	public	Agreement # 4600003005-A1	566811	2949208
Stormwater Treatment Area 2 (South Florida Water Management District)	public	Agreement # 4600003005-A1	545300	2919869
Stormwater Treatment Area 3 (South Florida Water Management District)	public	Agreement # 4600003005-A1	538471	2916480
Stormwater Treatment Area 5 (South Florida Water Management District)	public	Agreement # 4600003005-A1	508936	2925250
Stormwater Treatment Area 6 (South Florida Water Management District)	public	Agreement # 4600003005-A1	510385	2916070
Tiger Lake	public		464867	3085451
Water Conservation Area 2A	public		558729	2911050
Water Conservation Area 2B	public		562675	2895970
Water Conservation Area 3A south (north of Holiday Trail)	public		532100	2882860
Water Conservation Area 3A south (south of Holiday Trail)	public		523762	2861120
Water Conservation Area 3B	public		542610	2851370
