# Supplementary material for: Spatio-Temporal Variation in Age Structure and Abundance of the Endangered Snail Kite: Pooling across Regions Masks a Declining and Aging Population
Source: PLoS One. 2016 Sep 28;11(9):e0162690. doi: 10.1371/journal.pone.0162690 (PMC5040393; doi:10.1371/journal.pone.0162690)
Supplement: S2 Table — Model notation: “pent” = survey-specific probability of a snail kite entering the study area, “phi” = survey-specific probability that a snail kite remained in the study area, “p” = probability that a snail kite was detected in the study area given it was available, “.” = model parameter was assumed to be constant, “time” = model parameter varied among surveys, “Time” = model parameter varied as a linear function of time (by survey), “age” = model parameter varied between age class (0–1 years, 2–12 years, 13+ years), and “region” = model parameter varied for individuals in different regions. (PDF) [file pone.0162690.s006.pdf]

Year	Probability of entering study area (*pent*)	Probability of staying in study area (*phi*)	Probability of detection (*p*)	AICc	Delta AICc	AICc Weights	Num. Par
1997	survey (categorical)	survey (categorical)	region	492.03	0.00	0.41	14
1997	survey (linear)	age	region*survey (categorical)	492.04	0.01	0.41	19
1997	survey (categorical)	age	survey (categorical)	494.41	2.38	0.13	16
1997	survey (categorical)	age	region*survey (categorical)	497.08	5.05	0.03	22
1997	survey (linear)	survey (categorical)	region	498.87	6.84	0.01	11
1997	survey (categorical)	age*survey (categorical)	region	511.15	19.12	0.00	24
1997	survey (linear)+region	survey (categorical)*region	region	511.78	19.74	0.00	17
1997	survey (categorical)*region	survey (categorical)*region	region	511.95	19.92	0.00	24
1997	survey (linear)+region	age*survey (categorical)	region	515.67	23.64	0.00	22
1997	survey (linear)	constant	region	516.88	24.85	0.00	7
1997	survey (linear)+age	age*survey (categorical)	region	517.99	25.95	0.00	23
1997	age	age*survey (categorical)	region	518.14	26.11	0.00	22
1997	region	region	region	518.51	26.48	0.00	8
1997	survey (categorical)	constant	region	520.47	28.44	0.00	10
1997	age	region	region	520.63	28.60	0.00	9
1997	survey (linear)	age	region	520.72	28.69	0.00	9
1997	region	age	region	520.89	28.85	0.00	9
1997	survey (linear)	age	age	521.50	29.47	0.00	10
1997	age	age	region	523.02	30.98	0.00	10
1997	survey (categorical)	age	region	523.67	31.64	0.00	12
1997	survey (categorical)	age	age	525.72	33.68	0.00	13
1997	survey (categorical)*region	age*survey (categorical)	region	528.94	36.90	0.00	29
1997	age*region	age	region	529.48	37.45	0.00	13
1997	age*survey (categorical)	age*survey (categorical)	region	537.58	45.55	0.00	34

Year	Probability of entering study area (*pent*)	Probability of staying in study area (*phi*)	Probability of detection (*p*)	AICc	Delta AICc	AICc Weights	Num. Par
1998	survey (linear)	age	region*survey (categorical)	975.40	0.00	0.97	19
1998	survey (categorical)	age	region*survey (categorical)	982.46	7.06	0.03	22
1998	survey (linear)	age	region	989.72	14.32	0.00	9
1998	survey (linear)	age	age	989.92	14.51	0.00	10
1998	survey (categorical)	age	region	990.72	15.32	0.00	12
1998	survey (categorical)	age	age	991.12	15.72	0.00	13
1998	survey (linear)	survey (categorical)	region	991.71	16.31	0.00	11
1998	survey (categorical)	age	survey (categorical)	991.75	16.34	0.00	16
1998	survey (linear)+region	survey (categorical)*region	region	992.32	16.92	0.00	17

1998	survey (linear)	constant	region	994.81	19.41	0.00	7
1998	survey (categorical)	constant	region	995.89	20.49	0.00	10
1998	region	age	region	997.49	22.09	0.00	9
1998	age	age	region	999.56	24.16	0.00	10
1998	constant	constant	region	1001.12	25.72	0.00	6
1998	region	region	region	1002.26	26.85	0.00	8
1998	survey (categorical)	survey (categorical)	region	1002.29	26.89	0.00	14
1998	constant	survey (categorical)	region	1002.61	27.20	0.00	10
1998	age	region	region	1004.32	28.91	0.00	9
1998	survey (categorical)*region	age*survey (categorical)	region	1007.78	32.38	0.00	29
1998	survey (categorical)	age*survey (categorical)	region	1009.55	34.15	0.00	24
1998	survey (linear)+region	age*survey (categorical)	region	1011.00	35.60	0.00	22
1998	age	age*survey (categorical)	region	1017.02	41.61	0.00	22
1998	survey (categorical)*region	survey (categorical)*region	region	1017.75	42.35	0.00	24
1998	survey (linear)+age	age*survey (categorical)	region	1021.89	46.49	0.00	23

Year	Probability of entering study area (*pent*)	Probability of staying in study area (*phi*)	Probability of detection (*p*)	AICc	Delta AICc	AICc Weights	Num. Par
1999	survey (linear)	age	region*survey (categorical)	1358.08	0.00	0.90	19
1999	survey (linear)	constant	region	1363.72	5.64	0.05	5
1999	survey (categorical)	age	region*survey (categorical)	1364.14	6.05	0.04	22
1999	survey (linear)	age	region	1369.81	11.72	0.00	9
1999	survey (linear)	survey (categorical)	region	1371.90	13.81	0.00	11
1999	survey (categorical)	constant	region	1372.73	14.65	0.00	10
1999	survey (categorical)	age	region	1374.30	16.21	0.00	12
1999	survey (linear)+region	survey (categorical)*region	region	1377.15	19.06	0.00	17
1999	survey (linear)	age	age	1377.19	19.11	0.00	10
1999	survey (categorical)	age	survey (categorical)	1379.75	21.66	0.00	16
1999	survey (categorical)	age	age	1380.84	22.75	0.00	13
1999	survey (categorical)	survey (categorical)	region	1381.32	23.24	0.00	14
1999	region	region	region	1381.73	23.64	0.00	8
1999	age	region	region	1383.77	25.69	0.00	9
1999	constant	survey (categorical)	region	1386.64	28.56	0.00	10
1999	constant	constant	region	1388.85	30.77	0.00	6
1999	region	age	region	1393.85	35.76	0.00	9
1999	survey (linear)+region	age*survey (categorical)	region	1395.43	37.34	0.00	22
1999	age	age	region	1395.90	37.81	0.00	10
1999	survey (categorical)*region	survey (categorical)*region	region	1397.74	39.65	0.00	24
1999	survey (linear)+age	age*survey (categorical)	region	1400.83	42.75	0.00	23

1999	survey (categorical)	age*survey (categorical)	region	1401.96	43.87	0.00	24
1999	age*region	age	region	1402.08	43.99	0.00	13
1999	age	age*survey (categorical)	region	1411.75	53.66	0.00	22
1999	survey (categorical)*region	age*survey (categorical)	region	1432.88	74.80	0.00	29

Year	Probability of entering study area (*pent*)	Probability of staying in study area (*phi*)	Probability of detection (*p*)	AICc	Delta AICc	AICc Weights	Num. Par
2000	survey (linear)	constant	region	991.95	0.00	0.45	7
2000	survey (linear)	survey (categorical)	region	992.74	0.78	0.31	11
2000	survey (categorical)	survey (categorical)	region	994.93	2.97	0.10	14
2000	survey (linear)	age	region	995.60	3.65	0.07	9
2000	survey (categorical)	age	region	997.06	5.10	0.04	12
2000	survey (categorical)	constant	region	997.76	5.81	0.02	10
2000	survey (linear)+region	survey (categorical)*region	region	1002.08	10.13	0.00	17
2000	survey (linear)	age	region*survey (categorical)	1004.67	12.72	0.00	19
2000	survey (categorical)*region	survey (categorical)*region	region	1006.63	14.67	0.00	24
2000	survey (linear)	age	age	1006.86	14.91	0.00	10
2000	survey (linear)+region	age*survey (categorical)	region	1007.55	15.60	0.00	22
2000	survey (categorical)	age	age	1009.15	17.20	0.00	13
2000	survey (categorical)	age	region*survey (categorical)	1009.72	17.77	0.00	22
2000	survey (categorical)	age*survey (categorical)	region	1009.85	17.90	0.00	24
2000	survey (linear)+age	age*survey (categorical)	region	1010.84	18.89	0.00	23
2000	survey (categorical)	age	survey (categorical)	1012.44	20.49	0.00	16
2000	survey (categorical)*region	age*survey (categorical)	region	1014.36	22.40	0.00	29
2000	age*survey (categorical)	age*survey (categorical)	region	1028.04	36.09	0.00	34
2000	region	region	region	1031.34	39.39	0.00	8
2000	region	age	region	1033.04	41.09	0.00	9
2000	age	region	region	1033.40	41.45	0.00	9
2000	age	age	region	1035.12	43.16	0.00	10
2000	age*region	age	region	1041.37	49.42	0.00	13
2000	age	age*survey (categorical)	region	1042.11	50.16	0.00	22

Year	Probability of entering study area (*pent*)	Probability of staying in study area (*phi*)	Probability of detection (*p*)	AICc	Delta AICc	AICc Weights	Num. Par
2001	survey (linear)	age	region*survey (categorical)	859.26	0.00	0.97	19
2001	survey (categorical)	age	region*survey (categorical)	866.34	7.08	0.03	22

2001	survey (linear)+region	survey (categorical)*region	region	870.02	10.76	0.00	17
2001	survey (categorical)*region	survey (categorical)*region	region	884.86	25.60	0.00	24
2001	survey (linear)	survey (categorical)	region	894.60	35.34	0.00	11
2001	survey (categorical)	survey (categorical)	region	900.32	41.06	0.00	14
2001	survey (linear)	constant	region	904.02	44.76	0.00	7
2001	survey (categorical)	constant	region	904.96	45.70	0.00	10
2001	survey (linear)	age	region	907.73	48.47	0.00	9
2001	survey (categorical)	age	region	908.14	48.88	0.00	12
2001	survey (categorical)*region	age*survey (categorical)	region	910.13	50.87	0.00	29
2001	region	region	region	912.00	52.74	0.00	8
2001	survey (linear)+region	age*survey (categorical)	region	913.88	54.62	0.00	22
2001	age	region	region	914.07	54.81	0.00	9
2001	survey (linear)+age	age*survey (categorical)	region	920.44	61.18	0.00	23
2001	survey (categorical)	age*survey (categorical)	region	928.89	69.63	0.00	24
2001	age*survey (categorical)	age*survey (categorical)	region	932.82	73.56	0.00	34
2001	survey (categorical)	age	age	932.98	73.72	0.00	13
2001	survey (categorical)	age	survey (categorical)	938.12	78.86	0.00	16
2001	survey (linear)	age	age	941.31	82.05	0.00	10
2001	age	age*survey (categorical)	region	955.27	96.01	0.00	22
2001	region	age	region	957.12	97.86	0.00	9
2001	age	age	region	959.20	99.94	0.00	10
2001	age*region	age	region	965.47	106.21	0.00	13
2001	survey (categorical)	constant	region	997.76	138.50	0.00	10

**Year**	**Probability of entering study area (*pent*)**	**Probability of staying in study area (*phi*)**	**Probability of detection (*p*)**	**AICc**	**Delta AICc**	**AICc Weights**	**Num. Par**
2002	survey (linear)	constant	region	683.06	0.00	0.87	7
2002	survey (categorical)	constant	region	688.29	5.24	0.06	10
2002	survey (linear)	tsa	.	689.04	5.99	0.04	11
2002	survey (categorical)	survey (categorical)	region	691.44	8.38	0.01	13
2002	survey (categorical)	survey (categorical)	region	693.55	10.50	0.00	14
2002	survey (categorical)	constant	region	997.76	314.71	0.00	10

**Year**	**Probability of entering study area (*pent*)**	**Probability of staying in study area (*phi*)**	**Probability of detection (*p*)**	**AICc**	**Delta AICc**	**AICc Weights**	**Num. Par**
2003	survey (linear)	age	age	410.37	0.00	0.46	10
2003	survey (categorical)	constant	region	410.82	0.45	0.37	10
2003	survey (linear)	survey (categorical)	region	413.15	2.79	0.11	11
2003	survey (categorical)	age	region	415.16	4.79	0.04	12
2003	survey (categorical)	age	age	416.86	6.49	0.02	13
2003	survey (categorical)	survey (categorical)	region	419.73	9.36	0.00	14
2003	region	age	region	430.82	20.45	0.00	9

2003	survey (categorical)	age	region*survey (categorical)	432.05	21.68	0.00	22
2003	survey (linear)+region	age*survey (categorical)	region	437.92	27.55	0.00	22
2003	survey (categorical)	age*survey (categorical)	region	438.43	28.06	0.00	24
2003	age*region	age	region	439.50	29.13	0.00	13
2003	survey (linear)+age	age*survey (categorical)	region	440.20	29.83	0.00	23
2003	survey (categorical)*region	survey (categorical)*region	region	442.06	31.69	0.00	24
2003	survey (categorical)*region	age*survey (categorical)	region	450.72	40.35	0.00	29
2003	age*survey (categorical)	age*survey (categorical)	region	463.48	53.11	0.00	34
2003	age	age*survey (categorical)	region	758.94	348.58	0.00	22
2003	survey (categorical)	constant	region	997.76	587.39	0.00	10

**Year**	**Probability of entering study area (*pent*)**	**Probability of staying in study area (*phi*)**	**Probability of detection (*p*)**	**AICc**	**Delta AICc**	**AICc Weights**	**Num. Par**
2004	survey (linear)	age	region*survey (categorical)	662.07	0.00	0.88	19
2004	survey (categorical)	age	region*survey (categorical)	666.59	4.52	0.09	22
2004	survey (categorical)	constant	region	671.16	9.08	0.01	10
2004	survey (categorical)	age	region	671.53	9.46	0.01	12
2004	survey (categorical)	survey (categorical)	region	673.90	11.83	0.00	14
2004	survey (categorical)*region	survey (categorical)*region	region	675.10	13.02	0.00	24
2004	survey (linear)	constant	region	675.16	13.09	0.00	7
2004	survey (linear)	age	region	675.38	13.30	0.00	9
2004	survey (linear)	age	age	675.96	13.89	0.00	10
2004	survey (linear)+region	survey (categorical)*region	region	676.65	14.57	0.00	17
2004	survey (categorical)	age	age	677.50	15.43	0.00	13
2004	survey (linear)	survey (categorical)	region	678.16	16.08	0.00	11
2004	survey (categorical)	age	survey (categorical)	683.09	21.02	0.00	16
2004	survey (categorical)*region	age*survey (categorical)	region	685.16	23.08	0.00	29
2004	survey (categorical)	age*survey (categorical)	region	690.40	28.33	0.00	24
2004	survey (linear)+region	age*survey (categorical)	region	697.06	34.99	0.00	22
2004	region	region	region	697.66	35.59	0.00	8
2004	survey (linear)+age	age*survey (categorical)	region	698.95	36.88	0.00	23
2004	age	region	region	699.51	37.44	0.00	9
2004	constant	survey (categorical)	region	703.07	41.00	0.00	10
2004	constant	constant	region	705.17	43.10	0.00	6
2004	region	age	region	707.98	45.91	0.00	9

2004	age	age	region	710.42	48.35	0.00	10
2004	age*region	age	region	715.80	53.73	0.00	13
2004	age	age*survey (categorical)	region	724.07	61.99	0.00	22

**Year**	**Probability of entering study area (*pent*)**	**Probability of staying in study area (*phi*)**	**Probability of detection (*p*)**	**AICc**	**Delta AICc**	**AICc Weights**	**Num. Par**
2005	survey (linear)	age	region*survey (categorical)	891.04	0.00	1.00	19
2005	survey (linear)	age	region	945.97	54.93	0.00	9
2005	survey (linear)	constant	region	947.00	55.96	0.00	7
2005	survey (categorical)	age	region	953.56	62.52	0.00	12
2005	survey (categorical)	constant	region	953.82	62.78	0.00	10
2005	survey (linear)	age	age	953.85	62.81	0.00	10
2005	survey (categorical)	age	region*survey (categorical)	955.64	64.60	0.00	22
2005	survey (categorical)	survey (categorical)	region	956.54	65.50	0.00	14
2005	survey (categorical)	age	age	957.38	66.33	0.00	13
2005	survey (categorical)	age	survey (categorical)	957.71	66.67	0.00	16
2005	survey (linear)	survey (categorical)	region	961.07	70.03	0.00	11
2005	survey (linear)+region	survey (categorical)*region	region	972.06	81.02	0.00	17
2005	survey (linear)+region	age*survey (categorical)	region	981.06	90.02	0.00	22
2005	survey (categorical)	age*survey (categorical)	region	981.20	90.16	0.00	24
2005	survey (linear)+age	age*survey (categorical)	region	983.15	92.11	0.00	23
2005	survey (categorical)*region	survey (categorical)*region	region	987.14	96.10	0.00	24
2005	survey (categorical)*region	age*survey (categorical)	region	990.01	98.97	0.00	29
2005	region	age	region	1001.29	110.25	0.00	9
2005	region	region	region	1001.51	110.47	0.00	8
2005	age	age	region	1003.37	112.33	0.00	10
2005	age	region	region	1003.58	112.54	0.00	9
2005	constant	constant	region	1004.03	112.99	0.00	6
2005	constant	survey (categorical)	region	1008.83	117.79	0.00	10
2005	age*region	age	region	1009.66	118.62	0.00	13
2005	age	age*survey (categorical)	region	1046.90	155.86	0.00	22

**Year**	**Probability of entering study area (*pent*)**	**Probability of staying in study area (*phi*)**	**Probability of detection (*p*)**	**AICc**	**Delta AICc**	**AICc Weights**	**Num. Par**
2006	survey (linear)	age	region*survey (categorical)	676.91	0.00	1.00	19
2006	survey (categorical)	constant	region	734.65	57.74	0.00	10
2006	survey (linear)	constant	region	736.43	59.51	0.00	7
2006	survey (categorical)	age	region	737.78	60.86	0.00	12
2006	survey (categorical)	age	age	738.52	61.61	0.00	13
2006	survey (linear)	age	age	738.58	61.67	0.00	10

2006	survey (linear)	age	region	739.80	62.89	0.00	9
2006	survey (categorical)	age	region*survey (categorical)	741.70	64.78	0.00	22
2006	survey (categorical)	survey (categorical)	region	742.62	65.70	0.00	14
2006	survey (categorical)	age	survey (categorical)	745.73	68.82	0.00	16
2006	survey (linear)	survey (categorical)	region	751.03	74.12	0.00	11
2006	survey (linear)+region	survey (categorical)*region	region	765.24	88.33	0.00	17
2006	survey (categorical)	age*survey (categorical)	region	767.67	90.76	0.00	24
2006	constant	constant	region	769.72	92.80	0.00	6
2006	survey (categorical)*region	survey (categorical)*region	region	773.09	96.18	0.00	24
2006	region	age	region	773.49	96.58	0.00	9
2006	survey (linear)+region	age*survey (categorical)	region	773.49	96.58	0.00	22
2006	region	region	region	773.74	96.83	0.00	8
2006	survey (linear)+age	age*survey (categorical)	region	774.02	97.10	0.00	23
2006	age	age	region	775.60	98.68	0.00	10
2006	age	region	region	775.84	98.92	0.00	9
2006	constant	survey (categorical)	region	776.08	99.16	0.00	10
2006	age*region	age	region	781.98	105.06	0.00	13
2006	survey (categorical)*region	age*survey (categorical)	region	784.64	107.72	0.00	29
2006	age	age*survey (categorical)	region	801.00	124.08	0.00	22

**Year**	**Probability of entering study area (*pent*)**	**Probability of staying in study area (*phi*)**	**Probability of detection (*p*)**	**AICc**	**Delta AICc**	**AICc Weights**	**Num. Par**
2007	survey (linear)	age	region*survey (categorical)	669.99	0.00	0.95	19
2007	survey (categorical)	age	region*survey (categorical)	675.74	5.75	0.05	22
2007	survey (linear)	constant	region	693.68	23.69	0.00	7
2007	survey (linear)	age	region	694.45	24.46	0.00	9
2007	survey (categorical)	constant	region	700.06	30.07	0.00	10
2007	survey (linear)+region	survey (categorical)*region	region	700.48	30.49	0.00	17
2007	survey (categorical)*region	survey (categorical)*region	region	700.90	30.91	0.00	24
2007	survey (categorical)	age	region	701.58	31.58	0.00	12
2007	survey (linear)	age	age	707.86	37.87	0.00	10
2007	survey (categorical)	survey (categorical)	region	709.65	39.66	0.00	14
2007	survey (linear)	survey (categorical)	region	713.83	43.84	0.00	11
2007	survey (categorical)	age	survey (categorical)	716.52	46.53	0.00	16
2007	survey (categorical)	age	age	720.81	50.82	0.00	13
2007	survey (linear)+region	age*survey (categorical)	region	723.35	53.36	0.00	22
2007	survey (categorical)*region	age*survey (categorical)	region	738.83	68.84	0.00	29

2007	survey (categorical)	age*survey (categorical)	region	741.66	71.67	0.00	24
2007	region	region	region	746.06	76.07	0.00	8
2007	survey (linear)+age	age*survey (categorical)	region	746.39	76.40	0.00	23
2007	age	region	region	748.15	78.16	0.00	9
2007	region	age	region	789.44	119.45	0.00	9
2007	constant	constant	region	790.05	120.06	0.00	6
2007	age	age	region	791.54	121.55	0.00	10
2007	constant	survey (categorical)	region	796.95	126.96	0.00	10
2007	age*region	age	region	797.90	127.91	0.00	13
2007	age	age*survey (categorical)	region	824.47	154.47	0.00	22

**Year**	**Probability of entering study area (*pent*)**	**Probability of staying in study area (*phi*)**	**Probability of detection (*p*)**	**AICc**	**Delta AICc**	**AICc Weights**	**Num. Par**
2008	survey (linear)	constant	region	893.03	0.00	0.52	7
2008	survey (categorical)	constant	region	895.14	2.11	0.18	10
2008	survey (linear)	age	region	896.50	3.48	0.09	9
2008	survey (linear)	age	region*survey (categorical)	896.77	3.75	0.08	19
2008	survey (categorical)	survey (categorical)	region	897.91	4.88	0.05	14
2008	survey (categorical)	age	region	898.43	5.40	0.03	12
2008	survey (linear)	age	age	899.30	6.27	0.02	10
2008	survey (categorical)	age	region*survey (categorical)	899.97	6.94	0.02	22
2008	survey (categorical)	age	age	901.45	8.42	0.01	13
2008	survey (linear)	survey (categorical)	region	901.96	8.93	0.01	11
2008	survey (categorical)	age	survey (categorical)	904.40	11.38	0.00	16
2008	survey (linear)+region	survey (categorical)*region	region	918.30	25.28	0.00	17
2008	survey (categorical)	age*survey (categorical)	region	921.06	28.03	0.00	24
2008	survey (linear)+region	age*survey (categorical)	region	921.36	28.33	0.00	22
2008	survey (categorical)*region	survey (categorical)*region	region	921.96	28.94	0.00	24
2008	survey (linear)+age	age*survey (categorical)	region	925.43	32.40	0.00	23
2008	survey (categorical)*region	age*survey (categorical)	region	931.32	38.29	0.00	29
2008	region	region	region	993.04	100.02	0.00	8
2008	age	region	region	995.12	102.09	0.00	9
2008	constant	constant	region	997.02	104.00	0.00	6
2008	constant	survey (categorical)	region	997.20	104.18	0.00	10
2008	region	age	region	1000.53	107.50	0.00	9
2008	age	age	region	1002.61	109.58	0.00	10
2008	age*region	age	region	1008.90	115.88	0.00	13
2008	age	age*survey (categorical)	region	1019.98	126.95	0.00	22

Year	Probability of entering study area (*pent*)	Probability of staying in study area (*phi*)	Probability of detection (*p*)	AICc	Delta AICc	AICc Weights	Num. Par
2009	survey (linear)+age	age*survey (categorical)	region	963.84	0.00	0.61	16
2009	survey (linear)	age	region	965.37	1.53	0.28	9
2009	survey (linear)+region	survey (categorical)*region	region	968.71	4.88	0.05	17
2009	survey (linear)	constant	region	970.00	6.16	0.03	7
2009	survey (categorical)	age	region	970.80	6.96	0.02	12
2009	survey (linear)	survey (categorical)	region	973.14	9.30	0.01	11
2009	survey (linear)	age	region*survey (categorical)	974.64	10.80	0.00	19
2009	survey (categorical)	constant	region	975.38	11.54	0.00	10
2009	survey (linear)+region	age*survey (categorical)	region	978.07	14.23	0.00	22
2009	survey (categorical)	survey (categorical)	region	978.84	15.00	0.00	14
2009	survey (categorical)	age	region*survey (categorical)	979.10	15.26	0.00	22
2009	survey (categorical)*region	survey (categorical)*region	region	980.11	16.27	0.00	24
2009	survey (linear)	age	age	980.91	17.07	0.00	8
2009	survey (categorical)	age*survey (categorical)	region	982.30	18.46	0.00	24
2009	survey (categorical)*region	age*survey (categorical)	region	989.62	25.78	0.00	29
2009	survey (categorical)	age	age	990.68	26.85	0.00	13
2009	survey (categorical)	age	survey (categorical)	995.14	31.30	0.00	16
2009	age*survey (categorical)	age*survey (categorical)	region	998.91	35.07	0.00	34
2009	region	region	region	1031.50	67.66	0.00	8
2009	age	region	region	1033.56	69.72	0.00	9
2009	region	age	region	1043.48	79.64	0.00	9
2009	age	age	region	1045.55	81.72	0.00	10
2009	constant	constant	region	1047.25	83.42	0.00	6
2009	constant	survey (categorical)	region	1047.79	83.95	0.00	10
2009	age*region	age	region	1051.82	87.98	0.00	13
2009	age	age*survey (categorical)	region	1053.81	89.98	0.00	22

Year	Probability of entering study area (*pent*)	Probability of staying in study area (*phi*)	Probability of detection (*p*)	AICc	Delta AICc	AICc Weights	Num. Par
2010	survey (linear)	age	region*survey (categorical)	984.96	0.00	0.42	19
2010	survey (linear)	age	region	986.36	1.40	0.21	9
2010	survey (categorical)	age	region*survey (categorical)	987.10	2.14	0.14	22
2010	survey (linear)	age	age	987.49	2.53	0.12	10
2010	survey (categorical)	age	region	988.18	3.22	0.08	12
2010	survey (linear)	constant	region	992.38	7.42	0.01	7
2010	survey (categorical)	constant	region	992.79	7.83	0.01	10
2010	survey (linear)	survey (categorical)	region	998.74	13.77	0.00	11

2010	survey (categorical)	age	survey (categorical)	1000.14	15.18	0.00	16
2010	survey (categorical)	survey (categorical)	region	1001.38	16.42	0.00	14
2010	survey (linear)+region	survey (categorical)*region	region	1009.54	24.58	0.00	17
2010	survey (categorical)	age*survey (categorical)	region	1014.48	29.52	0.00	24
2010	survey (linear)+region	age*survey (categorical)	region	1018.61	33.65	0.00	22
2010	survey (categorical)*region	survey (categorical)*region	region	1020.28	35.31	0.00	24
2010	survey (linear)+age	age*survey (categorical)	region	1021.12	36.16	0.00	23
2010	survey (categorical)*region	age*survey (categorical)	region	1038.01	53.05	0.00	29
2010	region	age	region	1061.53	76.57	0.00	9
2010	age	age	region	1063.60	78.64	0.00	10
2010	constant	constant	region	1069.58	84.62	0.00	6
2010	age*region	age	region	1069.87	84.91	0.00	13
2010	constant	survey (categorical)	region	1077.17	92.21	0.00	10
2010	age	age*survey (categorical)	region	1085.01	100.05	0.00	22
2010	survey (categorical)	age	region*survey (categorical)	1366.07	381.11	0.00	22

**Year**	**Probability of entering study area (*pent*)**	**Probability of staying in study area (*phi*)**	**Probability of detection (*p*)**	**AICc**	**Delta AICc**	**AICc Weights**	**Num. Par**
2011	survey (linear)	age	region*survey (categorical)	1256.57	0.00	0.77	19
2011	survey (categorical)	age	region*survey (categorical)	1259.08	2.51	0.22	22
2011	survey (linear)	age	region	1266.13	9.56	0.01	9
2011	survey (categorical)	age	region	1266.18	9.61	0.01	12
2011	survey (categorical)	constant	region	1270.96	14.40	0.00	10
2011	survey (linear)	constant	region	1271.06	14.50	0.00	7
2011	survey (linear)+region	survey (categorical)*region	region	1273.56	16.99	0.00	17
2011	survey (linear)	age	age	1274.58	18.01	0.00	10
2011	survey (categorical)	age	age	1276.34	19.77	0.00	13
2011	survey (linear)	survey (categorical)	region	1276.58	20.01	0.00	11
2011	survey (categorical)	survey (categorical)	region	1279.79	23.23	0.00	14
2011	survey (linear)+region	age*survey (categorical)	region	1281.87	25.30	0.00	22
2011	survey (categorical)*region	survey (categorical)*region	region	1283.40	26.83	0.00	24
2011	survey (categorical)	age*survey (categorical)	region	1286.47	29.91	0.00	24
2011	survey (linear)+age	age*survey (categorical)	region	1288.16	31.59	0.00	23
2011	survey (categorical)*region	age*survey (categorical)	region	1293.24	36.67	0.00	29
2011	survey (categorical)	age	survey (categorical)	1295.38	38.81	0.00	16

2011	region	region	region	1434.90	178.33	0.00	8
2011	age	region	region	1436.95	180.38	0.00	9
2011	region	age	region	1456.19	199.63	0.00	9
2011	age	age	region	1458.25	201.68	0.00	10
2011	constant	constant	region	1462.56	206.00	0.00	6
2011	age*region	age	region	1464.44	207.87	0.00	13
2011	constant	survey (categorical)	region	1465.42	208.86	0.00	10
2011	age	age*survey (categorical)	region	1471.58	215.02	0.00	22

Year	Probability of entering study area (*pent*)	Probability of staying in study area (*phi*)	Probability of detection (*p*)	AICc	Delta AICc	AICc Weights	Num. Par
2012	survey (categorical)	age	region	2157.44	0.00	0.84	12
2012	survey (linear)	age	region*survey (categorical)	2161.66	4.22	0.10	19
2012	survey (linear)	age	age	2163.56	6.12	0.04	10
2012	survey (categorical)	age	age	2166.23	8.79	0.01	13
2012	survey (categorical)	age	region*survey (categorical)	2167.18	9.74	0.01	22
2012	survey (linear)	age	region	2168.51	11.07	0.00	9
2012	survey (categorical)	constant	region	2171.49	14.04	0.00	10
2012	survey (linear)	constant	region	2173.04	15.60	0.00	7
2012	survey (linear)+region	age*survey (categorical)	region	2174.90	17.46	0.00	22
2012	survey (linear)+region	survey (categorical)*region	region	2176.72	19.27	0.00	17
2012	survey (categorical)	survey (categorical)	region	2178.75	21.31	0.00	14
2012	survey (linear)	survey (categorical)	region	2179.65	22.20	0.00	11
2012	survey (categorical)	age*survey (categorical)	region	2180.72	23.27	0.00	24
2012	survey (categorical)	age	survey (categorical)	2184.72	27.28	0.00	16
2012	survey (categorical)*region	survey (categorical)*region	region	2194.06	36.62	0.00	24
2012	survey (categorical)*region	age*survey (categorical)	region	2198.36	40.92	0.00	29
2012	survey (linear)+age	age*survey (categorical)	region	2204.52	47.07	0.00	23
2012	region	age	region	2314.50	157.05	0.00	9
2012	age	age	region	2316.53	159.09	0.00	10
2012	region	region	region	2317.39	159.94	0.00	8
2012	age	region	region	2319.42	161.97	0.00	9
2012	constant	survey (categorical)	region	2322.18	164.73	0.00	10
2012	constant	constant	region	2322.46	165.02	0.00	6
2012	age*region	age	region	2322.66	165.21	0.00	13
2012	age	age*survey (categorical)	region	2331.06	173.61	0.00	22

Year	Probability of entering study area (*pent*)	Probability of staying in study area (*phi*)	Probability of detection (*p*)	AICc	Delta AICc	AICc Weights	Num. Par
2013	survey (categorical)	age	age	2720.51	0.00	1.00	13
2013	survey (linear)	age	age	2737.65	17.14	0.00	10

2013	survey (categorical)	age	region	2738.61	18.10	0.00	12
2013	survey (linear)	age	region*survey (categorical)	2746.57	26.05	0.00	19
2013	survey (categorical)	age	region*survey (categorical)	2754.53	34.01	0.00	22
2013	survey (categorical)	constant	region	2756.65	36.14	0.00	10
2013	survey (categorical)	age	survey (categorical)	2761.00	40.49	0.00	16
2013	survey (categorical)	age*survey (categorical)	region	2767.01	46.49	0.00	24
2013	survey (categorical)	survey (categorical)	region	2767.65	47.14	0.00	14
2013	survey (linear)	age	region	2769.36	48.85	0.00	9
2013	survey (linear)	constant	region	2769.59	49.08	0.00	7
2013	survey (categorical)*region	age*survey (categorical)	region	2777.82	57.31	0.00	29
2013	survey (linear)+region	age*survey (categorical)	region	2779.38	58.87	0.00	22
2013	survey (linear)	survey (categorical)	region	2782.55	62.03	0.00	11
2013	survey (categorical)*region	survey (categorical)*region	region	2783.08	62.56	0.00	24
2013	survey (linear)+region	survey (categorical)*region	region	2792.59	72.08	0.00	17
2013	survey (linear)+age	age*survey (categorical)	region	2794.53	74.01	0.00	23
2013	region	age	region	2987.39	266.88	0.00	9
2013	age	age	region	2989.42	268.90	0.00	10
2013	age*region	age	region	2995.51	275.00	0.00	13
2013	age	age*survey (categorical)	region	3002.99	282.48	0.00	22
2013	region	region	region	3006.71	286.20	0.00	8
2013	constant	survey (categorical)	region	3007.22	286.70	0.00	10
2013	constant	constant	region	3008.58	288.06	0.00	6
2013	age	region	region	3008.73	288.22	0.00	9
