# Supplementary material for: Spatio-Temporal Variation in Age Structure and Abundance of the Endangered Snail Kite: Pooling across Regions Masks a Declining and Aging Population
Source: PLoS One. 2016 Sep 28;11(9):e0162690. doi: 10.1371/journal.pone.0162690 (PMC5040393; doi:10.1371/journal.pone.0162690)
Supplement: S3 Table — Model notation: “pent” = survey-specific probability of a snail kite entering the study area, “phi” = survey-specific probability that a snail kite remained in the study area, “p” = probability that a snail kite was detected in the study area given it was available, “.” = model parameter was assumed to be constant, “time” = model parameter varied among surveys, “Time” = model parameter varied as a linear function of time (by survey), and “age” = model parameter varied between age class (0–1 years, 2–12 years, 13+ years). (PDF) [file pone.0162690.s007.pdf]

Year	Probability of entering study area (*pent*)	Probability of staying in study area (*phi*)	Probability of detection (*p*)	AICc	Delta AICc	AICc Weights	Num. Par
1997	survey (categorical)	constant	survey (categorical)	491.03	0.00	0.62	14
1997	age*survey (categorical)	constant	survey (categorical)	493.01	1.98	0.23	19
1997	survey (categorical)	age	survey (categorical)	494.41	3.38	0.11	16
1997	survey (linear)	survey (categorical)	constant	496.83	5.80	0.03	10
1997	age	survey (categorical)	constant	499.84	8.81	0.01	11
1997	age*survey (categorical)	survey (categorical)	constant	503.18	12.15	0.00	23
1997	survey (categorical)	age	constant	517.46	26.43	0.00	11
1997	constant	age	age	519.38	28.35	0.00	9
1997	age	age	age	523.65	32.62	0.00	11
1997	age*survey (categorical)	survey (categorical)	constant	525.51	34.48	0.00	19

Year	Probability of entering study area (*pent*)	Probability of staying in study area (*phi*)	Probability of detection (*p*)	AICc	Delta AICc	AICc Weights	Num. Par
1998	survey (linear)	age	constant	1000.49	0.00	0.70	8
1998	survey (categorical)	age	constant	1003.79	3.29	0.14	11
1998	survey (categorical)	survey (categorical)	constant	1005.69	5.19	0.05	13
1998	survey (linear)	constant	constant	1005.90	5.41	0.05	6
1998	survey (categorical)	constant	constant	1006.41	5.91	0.04	9
1998	survey (linear)	age * survey (categorical)	constant	1008.69	8.20	0.01	20
1998	survey (categorical)	age * survey (categorical)	constant	1009.32	8.83	0.01	23
1998	survey (linear) + age	age * survey (categorical)	constant	1010.77	10.28	0.00	22
1998	survey (categorical)	constant	survey (categorical)	1012.48	11.99	0.00	14
1998	survey (categorical)	age	survey (categorical)	1013.11	12.62	0.00	16
1998	survey (linear)	survey (categorical)	constant	1013.79	13.30	0.00	10
1998	age	age * survey (categorical)	constant	1016.21	15.72	0.00	21
1998	age	survey (categorical)	constant	1027.11	26.62	0.00	11
1998	age*survey (categorical)	survey (categorical)	constant	1029.64	29.15	0.00	19
1998	age*survey (categorical)	survey (categorical)	constant	1052.61	52.11	0.00	23
1998	age*survey (categorical)	constant	survey (categorical)	1443.35	442.86	0.00	24

Year	Probability of entering study area (*pent*)	Probability of staying in study area (*phi*)	Probability of detection (*p*)	AICc	Delta AICc	AICc Weights	Num. Par
1999	survey (linear)	constant	constant	1387.88	0.00	0.47	6
1999	survey (linear)	age	constant	1389.16	1.28	0.25	8
1999	survey (categorical)	survey (categorical)	constant	1391.06	3.18	0.10	11
1999	survey (linear)	survey (categorical)	constant	1391.56	3.68	0.07	10
1999	survey (categorical)	constant	constant	1391.81	3.93	0.07	9
1999	survey (linear)	age	constant	1392.58	4.70	0.04	11
1999	survey (categorical)	age	survey (categorical)	1401.65	13.77	0.00	16
1999	age	survey (categorical)	constant	1403.19	15.31	0.00	8
1999	constant	age	constant	1403.92	16.04	0.00	7
1999	age*survey (categorical)	constant	survey (categorical)	1404.53	16.65	0.00	24
1999	constant	age	age	1404.87	16.99	0.00	9
1999	age	constant	constant	1405.24	17.36	0.00	7
1999	survey (linear)	age * survey (categorical)	constant	1406.40	18.52	0.00	20
1999	age	age	age	1408.03	20.15	0.00	11
1999	survey (categorical)	age * survey (categorical)	constant	1409.70	21.82	0.00	23
1999	survey (linear) + age	age * survey (categorical)	constant	1410.96	23.08	0.00	22
1999	age*survey (categorical)	survey (categorical)	constant	1414.00	26.12	0.00	23
1999	age*survey (categorical)	survey (categorical)	constant	1414.35	26.47	0.00	19
1999	age	age * survey (categorical)	constant	1424.10	36.22	0.00	21

Year	Probability of entering study area (*pent*)	Probability of staying in study area (*phi*)	Probability of detection (*p*)	AICc	Delta AICc	AICc Weights	Num. Par
2000	survey (linear)	constant	constant	1010.82	0.00	0.61	6
2000	survey (categorical)	survey (categorical)	constant	1013.51	2.69	0.16	13
2000	survey (categorical)	constant	constant	1013.98	3.16	0.12	9
2000	survey (linear)	age	constant	1014.69	3.87	0.09	8
2000	survey (categorical)	constant	survey (categorical)	1017.57	6.75	0.02	14
2000	survey (categorical)	age	constant	1021.66	10.84	0.00	11
2000	survey (categorical)	age	survey (categorical)	1026.63	15.81	0.00	16
2000	survey (categorical)	age * survey (categorical)	constant	1027.87	17.05	0.00	23
2000	survey (linear)	age * survey (categorical)	constant	1030.56	19.74	0.00	20
2000	survey (linear) + age	age * survey (categorical)	constant	1030.71	19.89	0.00	22
2000	age*survey (categorical)	survey (categorical)	constant	1038.20	27.38	0.00	19
2000	age	survey (categorical)	constant	1048.23	37.41	0.00	11
2000	age	age	age	1048.94	38.12	0.00	7

2000	age*survey (categorical)	survey (categorical)	constant	1052.61	41.79	0.00	23
2000	constant	age	constant	1052.84	42.03	0.00	7
2000	age	constant	constant	1052.89	42.07	0.00	7
2000	constant	age	age	1053.41	42.59	0.00	9
2000	age	age * survey (categorical)	constant	1063.61	52.79	0.00	21
2000	age*survey (categorical)	constant	survey (categorical)	1443.35	432.53	0.00	24

**Year**	**Probability of entering study area (*pent*)**	**Probability of staying in study area (*phi*)**	**Probability of detection (*p*)**	**AICc**	**Delta AICc**	**AICc Weights**	**Num. Par**
2001	survey (categorical)	constant	constant	932.83	0.00	0.65	9
2001	survey (categorical)	age	constant	935.27	2.44	0.19	11
2001	survey (categorical)	survey (categorical)	constant	936.68	3.85	0.10	13
2001	survey (linear)	survey (categorical)	constant	938.53	5.70	0.04	10
2001	survey (categorical)	constant	survey (categorical)	941.57	8.74	0.01	14
2001	survey (linear)	constant	constant	942.09	9.26	0.01	6
2001	survey (categorical)	age	survey (categorical)	944.01	11.18	0.00	16
2001	survey (linear)	age	constant	944.82	11.99	0.00	8
2001	age*survey (categorical)	survey (categorical)	constant	945.41	12.58	0.00	19
2001	survey (categorical)	age * survey (categorical)	constant	954.07	21.24	0.00	23
2001	survey (linear)	age * survey (categorical)	constant	957.84	25.01	0.00	20
2001	survey (linear) + age	age * survey (categorical)	constant	963.20	30.37	0.00	22
2001	age	survey (categorical)	constant	983.26	50.43	0.00	11
2001	constant	age	constant	987.72	54.89	0.00	7
2001	age	constant	constant	988.48	55.65	0.00	7
2001	constant	age	age	991.11	58.28	0.00	9
2001	age	age	age	995.27	62.44	0.00	11
2001	age	age * survey (categorical)	constant	1001.56	68.73	0.00	21
2001	age*survey (categorical)	survey (categorical)	constant	1052.61	119.78	0.00	23
2001	age*survey (categorical)	constant	survey (categorical)	1443.35	510.52	0.00	24

**Year**	**Probability of entering study area (*pent*)**	**Probability of staying in study area (*phi*)**	**Probability of detection (*p*)**	**AICc**	**Delta AICc**	**AICc Weights**	**Num. Par**
2002	survey (linear)	constant	constant	689.04	0.00	0.44	6
2002	survey (linear)	survey (categorical)	constant	690.63	1.59	0.20	9
2002	survey (linear)	age	age	691.48	2.44	0.13	10
2002	survey (linear)	age	constant	691.94	2.90	0.10	8
2002	survey (linear) + age	constant	constant	693.13	4.09	0.06	8
2002	survey (categorical)	constant	constant	694.37	5.33	0.03	8
2002	survey (categorical)	survey (categorical)	constant	694.64	5.60	0.03	11
2002	survey (categorical)	age	constant	697.51	8.48	0.01	10
2002	survey (categorical)	age	survey (categorical)	699.85	10.81	0.00	14
2002	survey (linear)	age	survey (categorical)	699.94	10.90	0.00	14
2002	survey (linear)	age * survey (categorical)	constant	702.47	13.43	0.00	16
2002	age*survey (categorical)	survey (categorical)	constant	707.77	18.73	0.00	16
2002	survey (categorical)	age * survey (categorical)	constant	708.57	19.53	0.00	19
2002	age*survey (categorical)	constant	survey (categorical)	708.91	19.87	0.00	20
2002	age*survey (categorical)	survey (categorical)	constant	709.67	20.63	0.00	19
2002	age	constant	constant	734.40	45.36	0.00	7
2002	age	survey (categorical)	constant	734.87	45.84	0.00	10
2002	age	age * survey (categorical)	constant	746.38	57.34	0.00	18

**Year**	**Probability of entering study area (*pent*)**	**Probability of staying in study area (*phi*)**	**Probability of detection (*p*)**	**AICc**	**Delta AICc**	**AICc Weights**	**Num. Par**
2003	survey (linear)	constant	constant	406.32	0.00	0.40	6
2003	survey (linear)	age	constant	407.22	0.90	0.25	8
2003	survey (categorical)	constant	constant	408.42	2.10	0.14	7
2003	survey (categorical)	age	constant	409.28	2.97	0.09	9
2003	survey (linear)	survey (categorical)	constant	410.48	4.16	0.05	8
2003	survey (categorical)	survey (categorical)	constant	412.60	6.29	0.02	9
2003	survey (linear) + age	survey (categorical)	constant	413.24	6.92	0.01	10
2003	survey (linear)	age	survey (categorical)	413.31	6.99	0.01	11
2003	survey (categorical)	age	survey (categorical)	414.59	8.27	0.01	12
2003	survey (categorical)	constant	survey (categorical)	414.78	8.46	0.01	10
2003	survey (linear)	age * survey (categorical)	constant	414.80	8.48	0.01	14
2003	survey (categorical)	age * survey (categorical)	constant	416.79	10.48	0.00	15
2003	age*survey (categorical)	age	constant	417.27	10.96	0.00	15
2003	age*survey (categorical)	survey (categorical)	constant	422.32	16.00	0.00	15
2003	age*survey (categorical)	constant	survey (categorical)	422.63	16.31	0.00	16

Year	Probability of entering study area (*pent*)	Probability of staying in study area (*phi*)	Probability of detection (*p*)	AICc	Delta AICc	AICc Weights	Num. Par
2004	survey (linear)	age	constant	673.20	0.00	0.61	6
2004	survey (categorical)	constant	constant	676.37	3.17	0.12	9
2004	survey (linear)	constant	constant	676.84	3.64	0.10	6
2004	survey (linear)	age	constant	677.47	4.27	0.07	11
2004	survey (categorical)	age	constant	677.51	4.31	0.07	11
2004	survey (categorical)	survey (categorical)	constant	680.11	6.90	0.02	13
2004	survey (linear)	survey (categorical)	constant	681.49	8.29	0.01	10
2004	survey (categorical)	constant	survey (categorical)	688.07	14.87	0.00	14
2004	survey (categorical)	age	survey (categorical)	689.77	16.56	0.00	16
2004	age*survey (categorical)	survey (categorical)	constant	693.26	20.05	0.00	19
2004	survey (linear)	age * survey (categorical)	constant	697.25	24.05	0.00	20
2004	survey (categorical)	age * survey (categorical)	constant	700.11	26.91	0.00	23
2004	survey (linear) + age	age * survey (categorical)	constant	707.52	34.32	0.00	22
2004	constant	age	constant	708.26	35.05	0.00	7
2004	constant	age	age	709.27	36.07	0.00	9
2004	age	constant	constant	710.64	37.44	0.00	7
2004	age	age	age	713.50	40.30	0.00	11
2004	age	survey (categorical)	constant	714.10	40.90	0.00	11
2004	age	age * survey (categorical)	constant	726.37	53.16	0.00	21
2004	age*survey (categorical)	constant	survey (categorical)	827.75	154.55	0.00	24
2004	age*survey (categorical)	survey (categorical)	constant	1052.61	379.40	0.00	23

Year	Probability of entering study area (*pent*)	Probability of staying in study area (*phi*)	Probability of detection (*p*)	AICc	Delta AICc	AICc Weights	Num. Par
2005	survey (linear)	constant	constant	968.77	0.00	0.27	6
2005	survey (linear)	age	constant	968.80	0.03	0.27	11
2005	survey (categorical)	age	constant	968.80	0.03	0.27	11
2005	survey (linear)	survey (categorical)	constant	970.05	1.28	0.14	10
2005	survey (categorical)	constant	constant	973.45	4.68	0.03	9
2005	survey (categorical)	survey (categorical)	constant	974.55	5.78	0.02	13
2005	survey (categorical)	age	survey (categorical)	981.80	13.03	0.00	16
2005	survey (linear)	age * survey (categorical)	constant	982.96	14.19	0.00	20
2005	survey (categorical)	constant	survey (categorical)	984.00	15.23	0.00	14
2005	survey (categorical)	age * survey (categorical)	constant	986.38	17.61	0.00	23
2005	survey (linear) + age	age * survey (categorical)	constant	987.31	18.54	0.00	22
2005	age*survey (categorical)	survey (categorical)	constant	993.94	25.17	0.00	19
2005	age*survey (categorical)	constant	survey (categorical)	1003.16	34.39	0.00	24
2005	constant	age	constant	1041.02	72.25	0.00	7
2005	constant	age	age	1045.03	76.26	0.00	9
2005	age	age	age	1049.20	80.43	0.00	11
2005	age	survey (categorical)	constant	1049.75	80.98	0.00	11
2005	age	constant	constant	1050.38	81.61	0.00	7
2005	age*survey (categorical)	survey (categorical)	constant	1052.61	83.84	0.00	23
2005	age	age * survey (categorical)	constant	1062.16	93.39	0.00	21

Year	Probability of entering study area (*pent*)	Probability of staying in study area (*phi*)	Probability of detection (*p*)	AICc	Delta AICc	AICc Weights	Num. Par
2006	survey (categorical)	constant	constant	744.71	0.00	0.54	9
2006	survey (linear)	constant	constant	746.45	1.75	0.23	6
2006	survey (linear)	age	constant	747.79	3.09	0.12	11
2006	survey (categorical)	age	constant	747.82	3.11	0.11	11
2006	survey (linear)	survey (categorical)	constant	756.18	11.47	0.00	10
2006	survey (categorical)	survey (categorical)	constant	756.26	11.56	0.00	13
2006	survey (categorical)	age * survey (categorical)	constant	762.63	17.92	0.00	23
2006	age*survey (categorical)	survey (categorical)	constant	763.96	19.26	0.00	19
2006	survey (categorical)	constant	survey (categorical)	766.51	21.81	0.00	14
2006	survey (linear)	age * survey (categorical)	constant	768.05	23.35	0.00	20
2006	survey (categorical)	age	survey (categorical)	769.19	24.48	0.00	16
2006	age*survey (categorical)	survey (categorical)	constant	771.42	26.71	0.00	23
2006	survey (linear) + age	age * survey (categorical)	constant	776.36	31.66	0.00	22
2006	age*survey (categorical)	constant	survey (categorical)	784.52	39.82	0.00	24
2006	constant	age	constant	796.49	51.78	0.00	7
2006	age	constant	constant	797.13	52.42	0.00	7
2006	constant	age	age	799.10	54.39	0.00	9
2006	age	age	age	803.31	58.61	0.00	11
2006	age	survey (categorical)	constant	806.77	62.07	0.00	11
2006	age	age * survey (categorical)	constant	824.68	79.97	0.00	21

Year	Probability of entering study area (*pent*)	Probability of staying in study area (*phi*)	Probability of detection (*p*)	AICc	Delta AICc	AICc Weights	Num. Par
2007	survey (linear)	constant	constant	715.81	0.00	0.73	6
2007	survey (linear)	age	constant	718.81	2.99	0.16	11
2007	survey (categorical)	constant	constant	720.25	4.44	0.08	9
2007	survey (categorical)	age	constant	723.03	7.22	0.02	11
2007	survey (categorical)	survey (categorical)	constant	725.57	9.75	0.01	13
2007	survey (categorical)	constant	survey (categorical)	731.34	15.53	0.00	14
2007	survey (linear)	age * survey (categorical)	constant	732.81	17.00	0.00	20
2007	survey (categorical)	age * survey (categorical)	constant	735.82	20.01	0.00	23
2007	survey (linear) + age	age * survey (categorical)	constant	737.61	21.79	0.00	22
2007	age*survey (categorical)	survey (categorical)	constant	740.19	24.37	0.00	23
2007	survey (linear)	survey (categorical)	constant	744.74	28.93	0.00	10
2007	age*survey (categorical)	constant	constant	745.72	29.91	0.00	19
2007	survey (categorical)	age	survey (categorical)	778.57	62.76	0.00	16
2007	constant	age	constant	803.42	87.60	0.00	7
2007	constant	age	age	806.94	91.12	0.00	9
2007	age	constant	constant	810.20	94.38	0.00	7
2007	age	age	age	811.14	95.33	0.00	11
2007	age	survey (categorical)	constant	814.13	98.32	0.00	11
2007	age	age * survey (categorical)	constant	820.91	105.10	0.00	21

Year	Probability of entering study area (*pent*)	Probability of staying in study area (*phi*)	Probability of detection (*p*)	AICc	Delta AICc	AICc Weights	Num. Par
2008	survey (linear)	constant	constant	896.82	0.00	0.47	4
2008	survey (linear)	age * survey (categorical)	constant	898.32	1.50	0.22	12
2008	survey (linear) + age	age * survey (categorical)	constant	898.46	1.64	0.21	8
2008	survey (categorical)	constant	constant	902.32	5.49	0.03	9
2008	survey (linear)	age	constant	903.05	6.22	0.02	11
2008	survey (categorical)	constant	survey (categorical)	903.63	6.81	0.02	14
2008	survey (categorical)	age	constant	903.80	6.98	0.01	11
2008	survey (categorical)	age	survey (categorical)	904.41	7.59	0.01	16
2008	survey (linear)	survey (categorical)	constant	905.07	8.25	0.01	10
2008	survey (categorical)	survey (categorical)	constant	905.98	9.16	0.00	13
2008	survey (categorical)	age * survey (categorical)	constant	915.84	19.02	0.00	23
2008	age*survey (categorical)	survey (categorical)	constant	921.37	24.55	0.00	19
2008	age*survey (categorical)	constant	survey (categorical)	922.75	25.93	0.00	24
2008	age*survey (categorical)	survey (categorical)	constant	924.89	28.07	0.00	23
2008	constant	age	age	999.38	102.55	0.00	9
2008	constant	age	constant	1000.67	103.85	0.00	7
2008	age	age	age	1003.55	106.72	0.00	11
2008	age	constant	constant	1005.35	108.53	0.00	7
2008	age	survey (categorical)	constant	1008.13	111.30	0.00	11
2008	age	age * survey (categorical)	constant	1015.37	118.55	0.00	21

Year	Probability of entering study area (*pent*)	Probability of staying in study area (*phi*)	Probability of detection (*p*)	AICc	Delta AICc	AICc Weights	Num. Par
2009	survey (linear)	age	constant	986.78	0.00	0.53	11
2009	survey (categorical)	age	constant	987.55	0.77	0.36	11
2009	survey (linear)	constant	constant	990.32	3.54	0.09	6
2009	survey (linear)	survey (categorical)	constant	995.06	8.28	0.01	10
2009	survey (categorical)	age	survey (categorical)	995.68	8.90	0.01	16
2009	survey (categorical)	constant	constant	996.08	9.30	0.01	9
2009	survey (categorical)	age * survey (categorical)	constant	996.33	9.55	0.00	23
2009	survey (linear)	age * survey (categorical)	constant	1000.45	13.67	0.00	20
2009	survey (categorical)	survey (categorical)	constant	1001.11	14.33	0.00	13
2009	age*survey (categorical)	survey (categorical)	constant	1006.01	19.23	0.00	19
2009	survey (linear) + age	age * survey (categorical)	constant	1027.01	40.24	0.00	22
2009	constant	age	constant	1050.50	63.72	0.00	7
2009	age*survey (categorical)	survey (categorical)	constant	1052.61	65.83	0.00	23
2009	constant	age	age	1053.71	66.93	0.00	9
2009	age	age	age	1057.64	70.86	0.00	11
2009	age	constant	constant	1065.35	78.57	0.00	7
2009	age	survey (categorical)	constant	1069.20	82.42	0.00	11
2009	age	age * survey (categorical)	constant	1072.20	85.42	0.00	21
2009	age*survey (categorical)	constant	survey (categorical)	1443.35	456.58	0.00	24

Year	Probability of entering study area (*pent*)	Probability of staying in study area (*phi*)	Probability of detection (*p*)	AICc	Delta AICc	AICc Weights	Num. Par
2010	survey (categorical)	age	constant	999.40	0.00	0.45	11
2010	survey (linear)	age	constant	999.40	0.00	0.45	11
2010	survey (categorical)	age	survey (categorical)	1003.60	4.20	0.06	16
2010	survey (linear)	constant	constant	1004.61	5.22	0.03	6
2010	survey (categorical)	constant	constant	1007.89	8.49	0.01	9
2010	survey (linear)	survey (categorical)	constant	1009.88	10.48	0.00	10
2010	survey (categorical)	constant	survey (categorical)	1010.03	10.63	0.00	14
2010	survey (categorical)	survey (categorical)	constant	1013.66	14.26	0.00	13
2010	survey (categorical)	age * survey (categorical)	constant	1015.00	15.60	0.00	23
2010	age*survey (categorical)	survey (categorical)	constant	1021.21	21.81	0.00	19
2010	survey (linear)	age * survey (categorical)	constant	1029.02	29.62	0.00	20
2010	survey (linear) + age	age * survey (categorical)	constant	1029.08	29.69	0.00	22
2010	age*survey (categorical)	survey (categorical)	constant	1052.61	53.21	0.00	23
2010	constant	age	constant	1066.28	66.89	0.00	7
2010	constant	age	age	1066.29	66.89	0.00	9
2010	age	age	age	1070.13	70.73	0.00	11
2010	age	constant	constant	1084.67	85.27	0.00	7
2010	age	survey (categorical)	constant	1091.45	92.05	0.00	11
2010	age	age * survey (categorical)	constant	1100.32	100.92	0.00	21

Year	Probability of entering study area (*pent*)	Probability of staying in study area (*phi*)	Probability of detection (*p*)	AICc	Delta AICc	AICc Weights	Num. Par
2011	survey (linear)	age	constant	1291.38	0.00	0.79	11
2011	survey (categorical)	age	constant	1294.79	3.41	0.14	11
2011	survey (categorical)	age	survey (categorical)	1296.46	5.08	0.06	16
2011	survey (categorical)	age * survey (categorical)	constant	1302.04	10.66	0.00	23
2011	survey (categorical)	constant	constant	1303.54	12.16	0.00	9
2011	survey (linear)	constant	constant	1306.19	14.81	0.00	6
2011	survey (categorical)	survey (categorical)	constant	1306.22	14.84	0.00	13
2011	survey (linear)	survey (categorical)	constant	1309.80	18.42	0.00	10
2011	survey (linear)	age * survey (categorical)	constant	1311.83	20.45	0.00	20
2011	survey (linear) + age	age * survey (categorical)	constant	1314.20	22.82	0.00	22
2011	age*survey (categorical)	survey (categorical)	constant	1315.60	24.22	0.00	23
2011	age*survey (categorical)	survey (categorical)	constant	1316.27	24.89	0.00	19
2011	age*survey (categorical)	constant	survey (categorical)	1316.59	25.21	0.00	24
2011	age*survey (categorical)	constant	survey (categorical)	1317.89	26.51	0.00	24
2011	survey (linear)	age * survey (categorical)	constant	1319.22	27.84	0.00	20
2011	constant	age	age	1462.85	171.47	0.00	9

Year	Probability of entering study area (*pent*)	Probability of staying in study area (*phi*)	Probability of detection (*p*)	AICc	Delta AICc	AICc Weights	Num. Par
2012	survey (linear)	age	constant	2190.27	0.00	0.91	11
2012	survey (categorical)	age	constant	2195.21	4.94	0.08	11
2012	survey (linear)	constant	constant	2199.67	9.40	0.01	6
2012	survey (categorical)	constant	constant	2203.15	12.88	0.00	9
2012	survey (categorical)	survey (categorical)	constant	2210.29	20.02	0.00	13
2012	survey (linear)	survey (categorical)	constant	2215.38	25.11	0.00	10
2012	survey (categorical)	age * survey (categorical)	constant	2216.74	26.47	0.00	23
2012	survey (categorical)	age	survey (categorical)	2217.12	26.86	0.00	16
2012	age*survey (categorical)	constant	constant	2224.57	34.31	0.00	19
2012	survey (linear)	age * survey (categorical)	constant	2227.00	36.74	0.00	20
2012	survey (categorical)	constant	survey (categorical)	2230.07	39.80	0.00	14
2012	survey (linear) + age	age * survey (categorical)	constant	2233.34	43.08	0.00	22
2012	age*survey (categorical)	survey (categorical)	constant	2239.65	49.39	0.00	23
2012	constant	age	age	2340.37	150.10	0.00	9
2012	age	age	age	2344.44	154.17	0.00	11
2012	constant	age	constant	2349.84	159.57	0.00	7
2012	age	constant	constant	2368.06	177.79	0.00	7
2012	age	survey (categorical)	constant	2379.21	188.94	0.00	11
2012	age	age * survey (categorical)	constant	2392.12	201.85	0.00	21
2012	age*survey (categorical)	constant	survey (categorical)	3570.40	1380.13	0.00	24

Year	Probability of entering study area (*pent*)	Probability of staying in study area (*phi*)	Probability of detection (*p*)	AICc	Delta AICc	AICc Weights	Num. Par
2013	survey (linear)	age	constant	2751.06	0.00	0.98	11
2013	survey (categorical)	age	constant	2758.70	7.65	0.02	11
2013	survey (categorical)	constant	constant	2776.72	25.66	0.00	9

2013	survey (categorical)	age	survey (categorical)	2778.46	27.41	0.00	16
2013	survey (categorical)	survey (categorical)	constant	2780.92	29.86	0.00	13
2013	survey (linear)	constant	constant	2796.08	45.02	0.00	6
2013	age*survey (categorical)	survey (categorical)	constant	2796.45	45.39	0.00	19
2013	survey (categorical)	age * survey (categorical)	constant	2800.79	49.74	0.00	23
2013	age*survey (categorical)	survey (categorical)	constant	2814.15	63.09	0.00	23
2013	survey (categorical)	constant	survey (categorical)	2814.70	63.64	0.00	14
2013	survey (linear)	survey (categorical)	constant	2819.57	68.51	0.00	10
2013	survey (linear) + age	age * survey (categorical)	constant	2825.97	74.91	0.00	22
2013	survey (linear)	age * survey (categorical)	constant	2833.11	82.05	0.00	20
2013	constant	age	age	2981.79	230.73	0.00	9
2013	age	age	age	2985.84	234.79	0.00	11
2013	constant	age	constant	2992.33	241.27	0.00	7
2013	age	constant	constant	3028.18	277.13	0.00	7
2013	age	survey (categorical)	constant	3049.54	298.49	0.00	11
2013	age	age * survey (categorical)	constant	3053.86	302.80	0.00	21
2013	age*survey (categorical)	constant	survey (categorical)	3754.63	1003.57	0.00	24
